# Adapting the reverse pyramid airplane boarding method for social distancing in times of COVID-19

**DOI:** 10.1371/journal.pone.0242131

**Published:** 2020-11-04

**Authors:** R. John Milne, Liviu-Adrian Cotfas, Camelia Delcea, Liliana Crăciun, Anca-Gabriela Molănescu

**Affiliations:** 1 David D. Reh School of Business, Clarkson University, Potsdam, NY, United States of America; 2 Department of Economic Informatics and Cybernetics, Bucharest University of Economic Studies, Bucharest, Romania; 3 Department of Economics and Economic Policies, Bucharest University of Economic Studies, Bucharest, Romania; Tsinghua University, CHINA

## Abstract

Social distancing resulting from the new coronavirus (SARS-CoV2) has disrupted the airplane boarding process. Social distancing norms reduce airplane capacity by keeping the middle seats unoccupied, while an imposed aisle social distance between boarding passengers slows the boarding. Recent literature suggests the Reverse Pyramid boarding method is a promising way to reduce health risk and keep boarding times low when 10 apron buses (essentially 10 boarding groups) are used to transport passengers from the airport terminal to a two-door airplane. We adapt the Reverse Pyramid method for social distancing when an airplane is boarded using a jet bridge that connects the terminal the airplane’s front door. We vary the number of boarding groups from two to six and use stochastic simulation and agent-based modelling to show the resulting impact on four performance evaluation metrics. Increasing the number of boarding groups from two to six reduces boarding time only up to four groups but continues to reduce infection risk up to six groups. If the passengers carry fewer luggage aboard the airplane, health risks (as well as boarding times) decrease. One adaptation of the Reverse Pyramid (RP) method (RP-Spread) provides slightly faster boarding times than the other (RP-Steep), when luggage volumes are high, while RP-Steep results in less risk to window seat passengers from later-boarding passengers walking by their row. Increasing the minimum aisle social distance from 1 m to 2 m increases boarding times but results in lower health risks to passengers walking down the aisle and to the previously seated passengers they pass.

## Introduction

The novel coronavirus SARS-CoV2 has produced a series of changes worldwide in different economic areas, businesses, and people’s personal lives. The global economy is projected to contract with worse negative effects than those generated by the 2008–2009 financial crisis [1]. According to Taylor [2], airplane transportation is one of the main sectors affected by the coronavirus outbreak, having multiple adverse effects related to both the passengers’ reluctance to engage in air travel and limitations imposed by governments, for instance, prohibiting international flights between some countries. The International Civil Aviation Organization (ICAO) expects an unprecedent drop in air travel demand [1], while in some regions the volume of air traffic dropped more than 90% because of the pandemic [3].

Over time, some international flights have been re-established among regions with relatively few infections [3]. For example, in Europe, the number of flights has increased in June 2020 by up to 400% compared with May 2020 [4].

Social distancing is one of the most discussed measures for reducing COVID-19 contagion during airplane travelling [5–7]. To ensure social distancing, airlines and airports have created a series of policies for passenger boarding including: boarding first passengers with seats in the rear rows of the airplane [8, 9], boarding using groups of 10 passengers [10], boarding based on passengers’ seat numbers [11], keeping the middle seats unoccupied [12], passengers not bringing carry-on luggage into the airplane cabin [13, 14], suspending priority boarding [14], using jet bridges when possible [14], limiting the number of passengers when apron buses are used [14], keeping a social distance of 1 m [14], and using masks and changing them every four hours [14]. Recent research investigates the assignment of passengers to seats on an airplane to improve social distancing among seated passengers [15]. Transport associations and agencies, such as the International Air Transport Association (IATA) and European Union Aviation Safety Agency (EASA), recommend that airplane operators ensure a physical distance among passengers and, where possible, that this distance be 1–2 m [16, 17]. The World Travel and Tourism Council recommends that airlines consider alternative boarding processes, such as back-to-front and window-to-aisle, and use an orderly boarding process for reducing the physical contact between passengers [18].

Researchers have investigated the connection between disease spreading and air transportation. Islam et al. [19] asserts that keeping middle seats unoccupied results in a substantial reduction in disease exposure. A recent paper by Derjany et al. [20] addresses the design of queues to mitigate infectious disease spread. They show that there is a strong correlation between contact rates and infection spread during epidemics.

Considering the research literature related to airplane boarding, a series of boarding methods have been proposed and implemented over time with the purpose of providing a faster boarding process that benefits the airlines and their passengers [21–24]. Most of the methods have been created for the case in which a jet bridge is used [21, 23, 25–32] and relatively few for the apron buses case [31–37]. Different assumptions accompany the methods, varying from the airplane characteristics [24–26, 38, 39], airplane occupancy rates [24, 25, 27, 28, 37, 40–42], passengers rules of movement and personal characteristics [23, 43], the presence of hand luggage [23, 25, 27, 29, 40, 44, 45], disturbances and passengers interferences [26, 29, 38, 41, 46–49]. Modeling passenger behavior has been made using a series of approaches, featuring the use of linear and mixed integer programming [30, 44, 46], genetic algorithms [26], pedestrian flow modeling [19, 20, 50], stochastic modeling [51–53], simulated annealing [54], grid-based simulation modelling [49], agent-based modelling [33, 55–58], empirical tests of the performance of the considered boarding methods [24], etc.

The Reverse Pyramid boarding method was developed by Van den Briel et al. [28]. This method segregates passengers into boarding groups depending on their airplane seats’ positions [56]. The boarding groups are created using a “diagonally load” scheme. This means that most groups will have some passengers with seats towards the rear of the airplane, while the other passengers within the same group will have seats in the middle (or front) of the airplane that are closer to the window than the seats of those passengers in the group seated near the rear of the airplane [31]. Within each boarding group, passengers enter the airplane in a random manner [59].

A recent paper [60] adapted classical boarding methods for social distancing conditions when 10 apron bus trips (corresponding to 10 boarding groups) transport passengers from the airport terminal to a two-door airplane. The authors evaluate the boarding methods according to the same four performance metrics we use in this paper. One of the metrics is the time to complete boarding of the airplane, and the other three metrics pertain to potential exposure to the novel coronavirus during boarding. One potential exposure (seat interferences) results from a seated passenger needing to stand to clear space for a later boarding window seat passenger. The other two infection risks pertain to later boarding passengers traversing the aisle passing (and thus potentially shedding the virus onto) passengers seated in aisle seats and window seats. If the risk to previously seated window seat passengers is not important and the boarding time is important for an airline, then a good choice would be to use the Reverse Pyramid—Spread method, which has the shortest boarding times and the best results for the other two health metrics. If the risk to window seat passengers becoming infected by later-boarding passengers is significant, then the Reverse Pyramid–Steep method would be the best choice because it is safer for window seat passengers and its boarding times are nearly as fast as those resulting from the Reverse Pyramid–Spread method. Of the methods tested by [60], the Reverse Pyramid methods had the best overall health risks—safer than the other methods—and the best boarding times.

The Reverse Pyramid–Spread and Reverse Pyramid–Steep methods were designed for use with 10 apron bus trips and a two-door airplane [60] under social distance conditions. We generalize the approach of these two methods to work for a general number of boarding groups (between two and six) for use with a jet-bridge connecting the airport terminal to the front door of the airplane. We test the generalized Reverse Pyramid–Spread and Reverse Pyramid–Steep approaches under social distance conditions. In these conditions, the middle seats of the airplane are unoccupied, and a minimum distance between boarding passengers advancing down the airplane’s aisle must be maintained.

We evaluate the boarding methods as a function of that aisle social distance (being 1 m or 2 m), the amount of luggage carried by passengers aboard the airplane, and the number of boarding groups. When using a jet bridge, airlines typically call, in turn, groups of passengers of a given priority class to board the airplane. In this paper, we vary the number of boarding groups from two to six, and provide numerical results for four performance metrics (as suggested in [61]) for the adapted Reverse Pyramid methods. The results provide insight for airline managers as they decide upon the following policy alternatives:

- the number of boarding groups of passengers to be called to board the airplane
- the boarding method (Reverse Pyramid–Spread or Reverse Pyramid–Steep)
- carry-on luggage restrictions
- the aisle social distance they recommend to their passengers

## Methods

**Fig 1:**
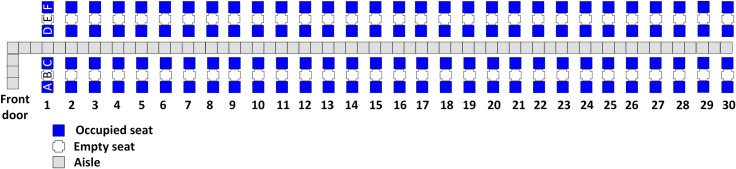
Airplane configuration.

Our methods are designed to work for a single-door Airbus A320 configuration with one aisle, thirty rows, and three seats on each side of the aisle as suggested by [61–63]. We assume that the middle seats have been blocked to preserve social distancing between seated passengers. The “middle seat empty” assumption used in this research is in line with the measures taken in practice by some of the airlines such as Delta [64] and Japan Airlines [65] during the coronavirus outbreak. In a recent preprint, Barnett [66] shows that with the “middle seat empty” policy, the risk of contracting COVID-19 from a nearby passenger is reduced in half when compared to a policy that assigns passengers to the middle seats. As a result of the “middle seat empty” policy, in our model, a full flight boards 120 passengers. Passengers enter the airplane through the front door, with the airplane configuration illustrated in Fig 1.

### Proposed boarding methods

With the proposed boarding methods, we adapt the Reverse Pyramid (RP) method to work with social distancing and a number of passenger groups varying between two and six. We assume that each boarding group contains the same number of passengers.

**Fig 2:**
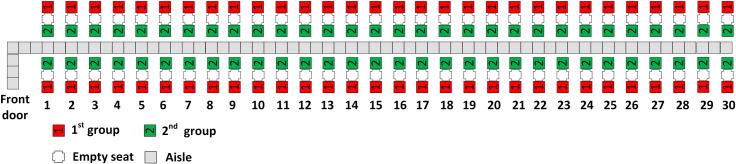
RP with 2 boarding groups.

With RP, the first group to board contains those passengers with seats closest to the rear of the airplane, and the final group to board contains those passengers with seats closest to the front door of the airplane. With two boarding groups, this results in the first group consisting of the window seat passengers and the second group consisting of the aisle seat passengers as illustrated in Fig 2. This is essentially the classical WilMA (Window-Middle-Aisle) boarding method, except adapted for social distancing as in [61].

**Fig 3:**
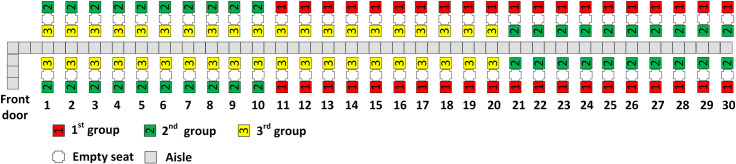
RP with 3 boarding groups.

With three boarding groups, there are 40 passengers in each group. RP assigns the 40 passengers with window seats closest to the rear of the airplane to the first group and the 40 passengers with aisle seats closest to the front of the airplane to the third (final) group. This results in the second boarding group having half of its passengers in aisle seats in rows 21–30 of the airplane and the other half of its passengers having window seats in rows 1–10. The resulting scheme for three boarding groups is illustrated in Fig 3.

When there are four or more boarding groups, the situation becomes more complicated as there are now two (or more) discretionary boarding groups—groups other than the first and final groups—that will have some passengers with window seats and the other passengers with aisle seats. We refer to these as *discretionary* groups because the classical RP method is unclear over how many of each seat type to contain in the discretionary boarding groups when social distancing applies. Our approach follows three principles for the assignment of aisle seat passengers to discretionary groups. First, the aisle seat passengers board the airplane in a back to front sequence. That is, the aisle seat passengers of discretionary boarding group *K—*1 have seats closer to the rear of the airplane than the aisle seat passengers of boarding group *K*. For example, the aisle seat passengers of the second group to board have seats closer to the rear door than those of the third group to board. The second principle ensures that both pairs of aisle seat passengers sitting in a same row are assigned to the same boarding group. The third principle is to assign an equal number of aisle seat passengers to each discretionary group. When the principles conflict, the first principle takes priority over the second principle, which takes priority over the third principle. When a conflict prevents the third principle from being applied, nearly equal numbers of aisle seat passengers are assigned to each group. The first two principles were established in [60], while the third principle is new with this manuscript. All three principles reflect design choices that minimize the number of times later-boarding passengers pass (and thereby possibly infect) previously seated passengers who are occupying the nearby aisle seats. Regarding that risk, we note that COVID-19 droplets and aerosols fall after expiration from a contagious walking passenger.

With those three principles determining the assignment of aisle seat passengers to boarding groups, the next decision is how to assign window seat passengers to boarding groups. Given our assumption of equal size boarding groups, the number of window seat passengers to assign to a boarding group is simply the number of passengers traveling in the boarding group minus its number of aisle seat passengers. With the Reverse Pyramid–Steep method (RP-Steep), the window seat passengers are assigned to groups in a back to front sequence.

**Fig 4:**
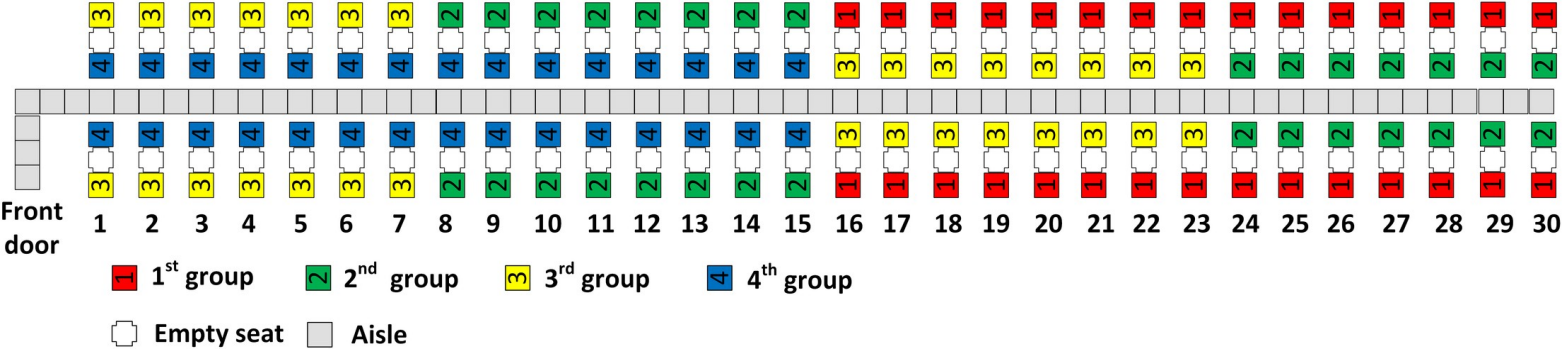
RP-Steep with 4 boarding groups.

The RP-Steep method with four boarding groups is illustrated in Fig 4. Observe that the window seat passengers are assigned to groups in a back to front sequence with the window seat passengers from group 1 in the rear half of the airplane, the group 3 window seat passengers in the rows closest to the front door of the airplane, and the group 2 window seat passengers seated between passengers of the first and third groups. Observe as well in Fig 4 that the first and second aisle seat assignment principles are followed. The aisle seat passengers are assigned in a back to front sequence and the two aisle seat passengers of every row are assigned to the same boarding group. Because the third aisle seat assignment principle conflicts with the second principle, nearly equal number of aisle seat passengers (7 and 8) are assigned to the second and third boarding groups respectively.

**Fig 5:**
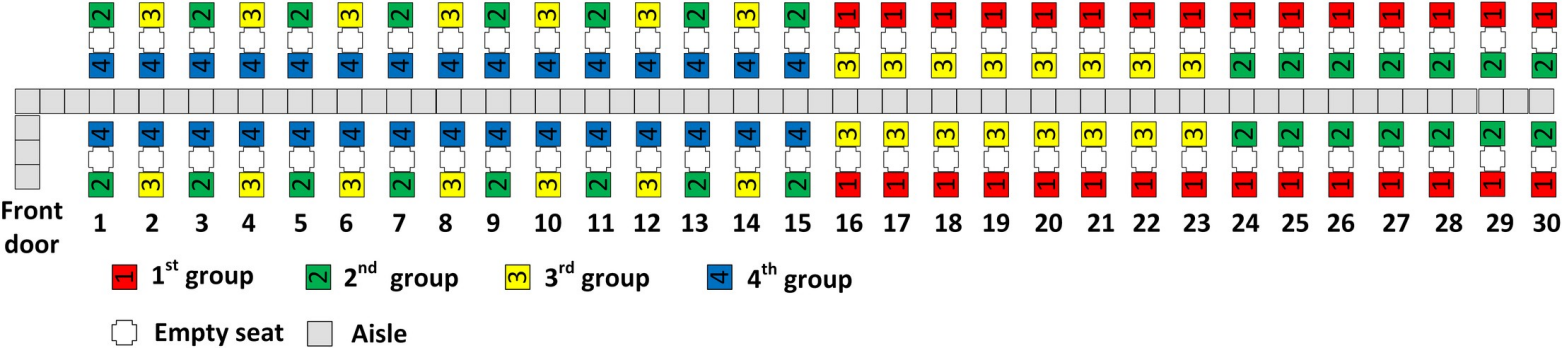
RP-Spread with 4 boarding groups.

The Reverse Pyramid–Spread (RP-Spread) method applies the same aisle seat principles as RP-Steep. However, RP-Spread assigns passengers to discretionary boarding groups so that each of the group’s window seat passengers are spread across the airplane. For instance, with a four person boarding group, the discretionary boarding groups 2 and 3 have window seat passengers in every other row respectively in rows 1–15 as illustrated in Fig 5.

**Fig 6:**
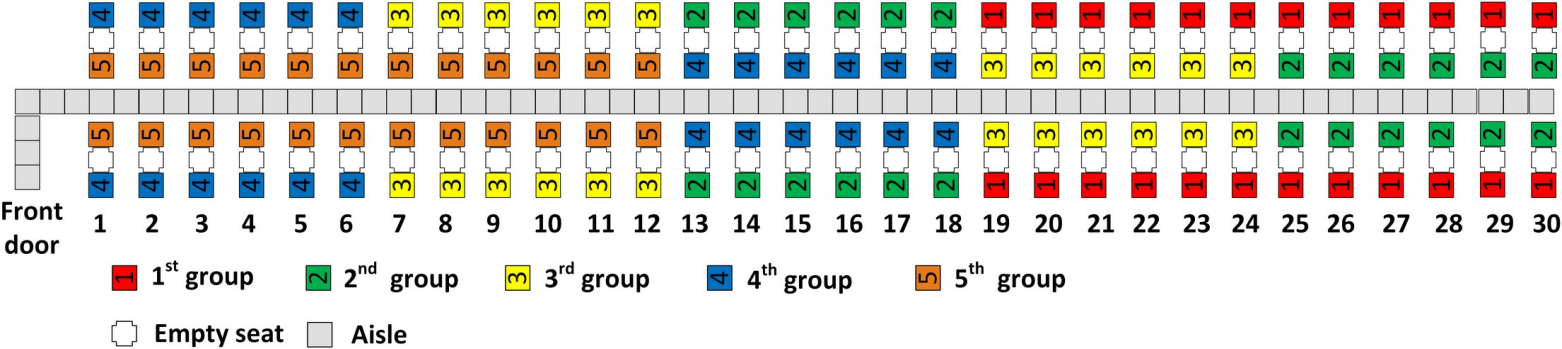
RP-Steep with 5 boarding groups.

**Fig 7:**
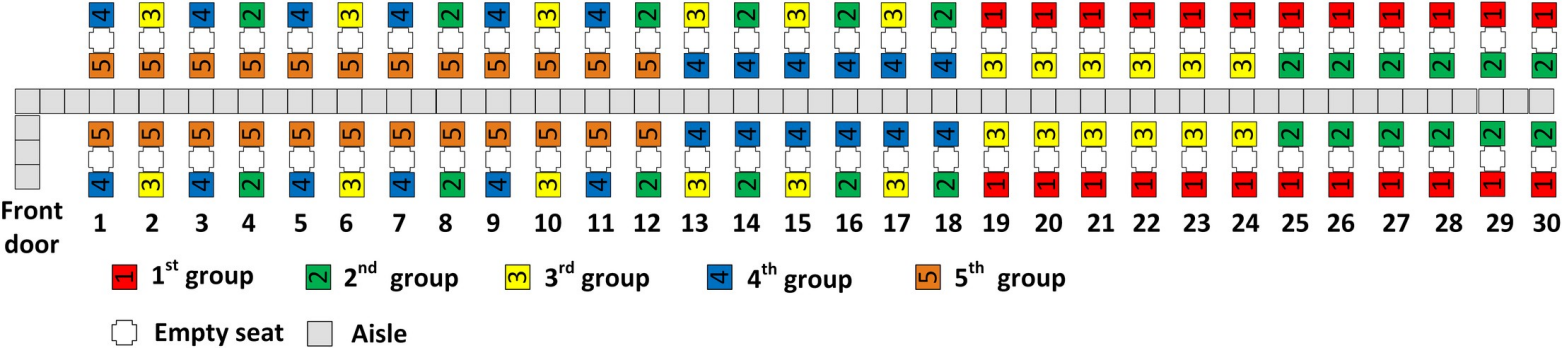
RP-Spread with 5 boarding groups.

**Fig 8:**
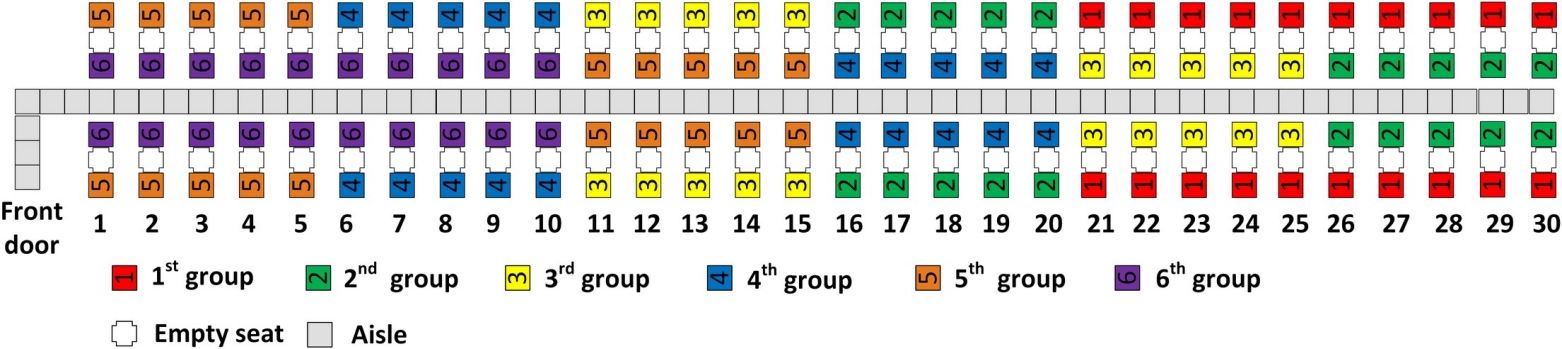
RP-Steep with 6 boarding groups.

**Fig 9:**
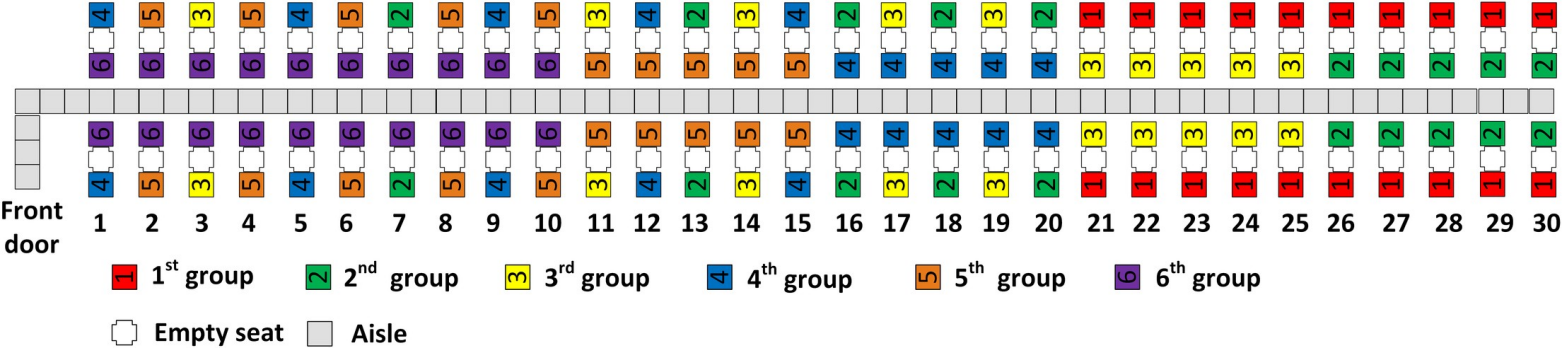
RP-Spread with 6 boarding groups.

With five and six boarding groups, all three aisle seat principles are fulfilled as illustrated in Figs 6 through 9. With more boarding groups than earlier, there are fewer passengers per group, but the general pattern of the RP-Steep and RP-Spread methods remain as before. Figs 6 and 8 illustrate the boarding scheme for RP-Steep for five and six boarding groups respectively. Figs 7 and 9 illustrate the boarding scheme for RP-Spread for five and six boarding groups respectively.

Having the boarding schemes to be tested for groups varying from two to six, an agent-based model in NetLogo 6.1.1 [67] has been created for facilitating their simulation and testing under the same initial conditions.

### Agent-based model description

Agent-based modeling is one of the most employed techniques for representing different phenomena in terms of agents and their interactions [68–70]. Human behavior is not exempt from the situations in which the agent-based modeling approach can be successfully applied due to the characteristics and the advantages offered by this type of modeling [71]. In the particular case of airplane boarding methods, the mobility of passengers heading towards their assigned seats and the design of the airplane cabin interior has been made using the NetLogo 6.1.1 platform [67]. This platform is popular among researchers due to the integrated visual interface (called “the world”), the intuitive and easy to write programming language, the existence of different types of agents, the possibility to have real-time graphics for analyzing variables, its integration with other specialized software programs, and the real-time user access to the state of any agent [35].

**Fig 10:**
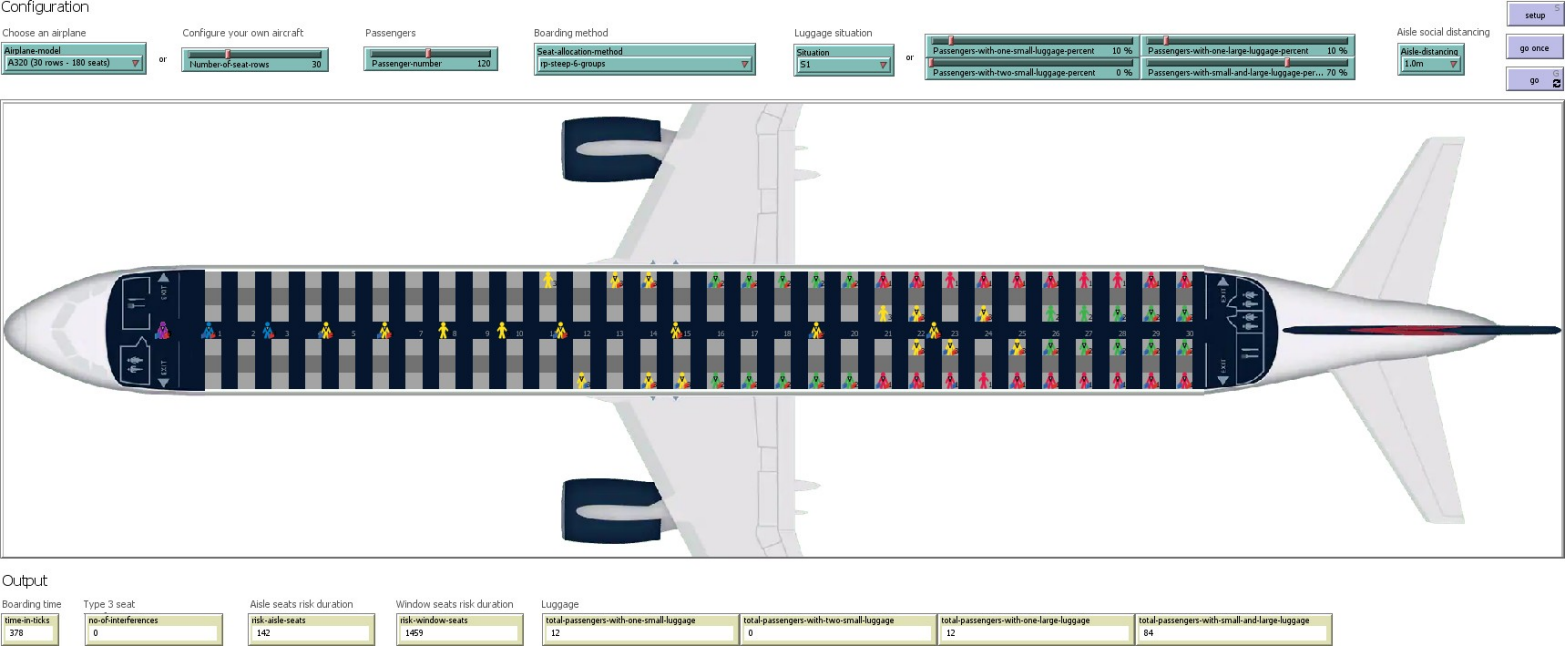
The GUI for the agent-based model in NetLogo 6.1.1 (example view for the RP-Steep method with 6 groups and 1 m aisle social distance).

The model’s graphical user interface (GUI) is presented in Fig 10. It contains three major areas. The area located in the upper portion of the screen enables a user to select the type of airplane, the number of passengers, the quantity of luggage carried inside the airplane, and the minimum aisle social distance to be kept among the agents. Three buttons (*setup*, *go*, and *go once*) are available near the top right of the screen. The central area presents the dynamic movement of the passengers heading towards their assigned seats during a simulation of the boarding process. The output area is located in the lower portion of the screen. A series of metrics are listed in the output area, as discussed in the following section.

**Fig 11:**
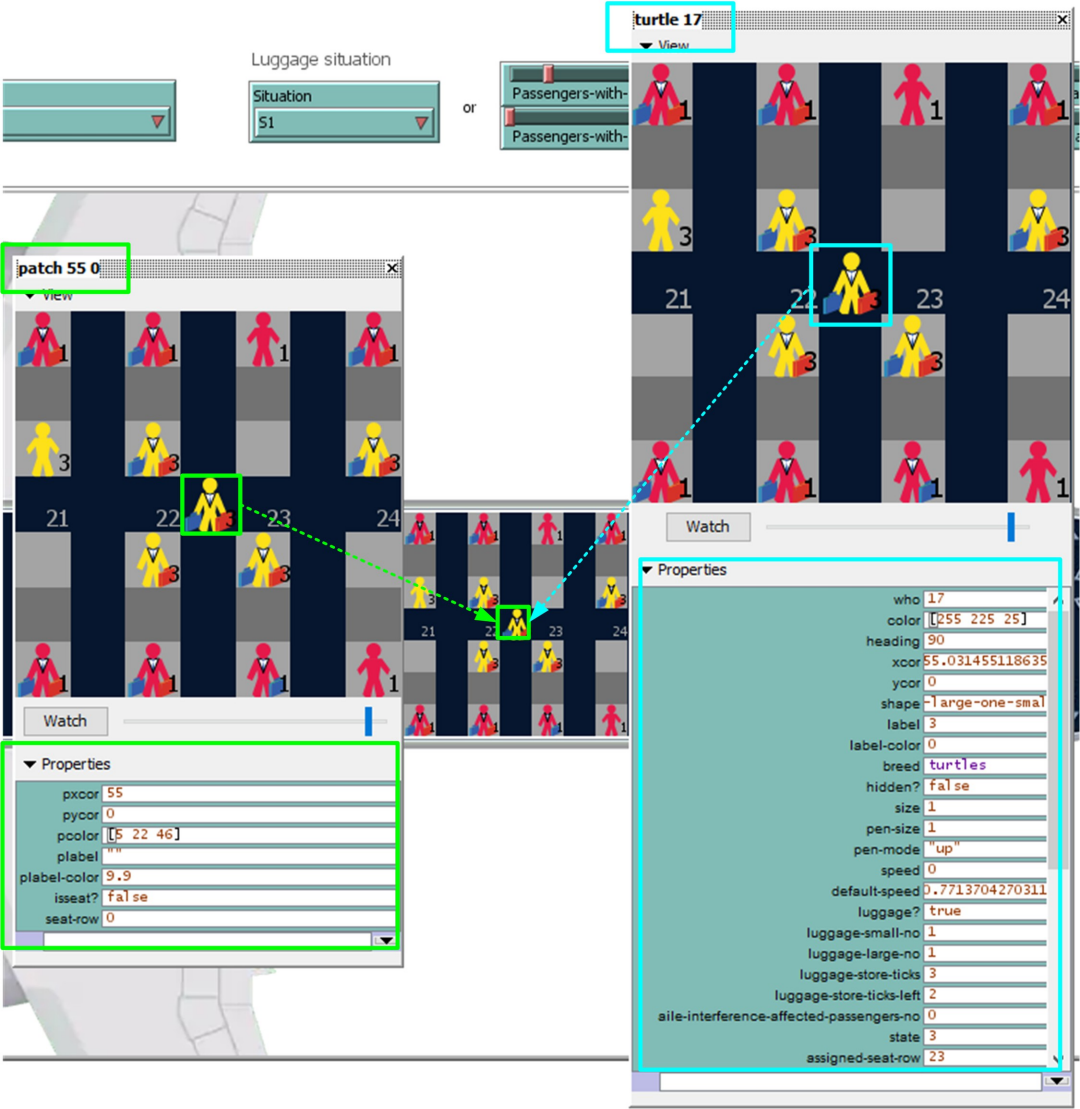
Example of a turtle agent (turtle 17) and a patch agent (patch 55 0).

To represent passengers and the airplane environment, we use two types of agents: “patches” and “turtles” as illustrated in Fig 11. The patches agents are small square pieces of ground with different attributes used for drawing the interior of the airplane, each of them representing a real surface equal to 0.4 m x 0.4 m as suggested by [62, 72]. The turtles agents are represented using the shape of a person carrying a particular amount of luggage (Fig 11) and their attributes have been chosen by considering the research literature for modeling the behavior of passengers inside an airplane [61].

While a series of agents’ properties have been chosen to offer a pleasant graphical interface, such as the colors of the aisle, seats, and passengers, there are a series of properties that are needed for a proper shaping of the agents’ behavior and characteristics. In the case of the patch agents, the only property worth mentioning here is the *isseat*? variable which can take a true or false value and facilitates movement of the turtle agents within the airplane. When true, the value of the *seat-row* variable takes integer numerical values between 1 and the number of rows of the airplane (in our case 30).

The main characteristics of the turtle agents and their descriptions are presented in Table 1.

**Table 1:** The main characteristics of the turtle agents.

Variable name	Description
*state*	This variable indicates the current state of the passenger. The states of a passenger during the boarding process are: boarded (while moving down the aisle), storing luggage, seating (while taking his/her seat), and seated.
*speed*	This represents the current speed of the turtle agent. If the agent is seated, the speed is 0. The speed can also be 0 if the agent is prevented from moving by another agent in front of it storing luggage. The maximum value for the speed is 1 patch / tick, where the tick is the time moment in NetLogo. This is equivalent to 0.33 m/s as suggested by [30, 51, 62].
*default-speed*	The values of this indicator depends on the number and type of luggage carried by the agent inside the airplane. When the agent has no luggage, the value can be up to 1 patch/tick, and with luggage, the value is between 0.6 patch / tick and 0.9 patch / tick [36]. A uniform probability distribution is used by the stochastic simulation model to generate the default-speed.
*luggage*?	This variable indicates whether the agent carries hand luggage inside the airplane.
*luggage-small-no*	This is the number of small luggage the agent brings inside the airplane. It can take 0, 1 or 2 as values.
*luggage-large-no*	This is the number of large luggage the agent brings inside the airplane. It can be either 0 or 1.
*luggage-store-ticks*	This variable is the number of ticks needed for an agent to store the hand luggage. It can take values between 0 (the passenger does not have hand luggage) and 6 ticks as determined based on the formula suggested by [73] and used by [25, 30, 33, 35, 55]. The formula is presented in Eq (1).
*luggage-store-ticks-left*	At each moment, this variable contains the remaining number of *luggage-store-ticks* needed to complete the storing of luggage in the overhead compartment.
*assigned-seat-row*	This is the number of the row in which the agent has an assigned seat.
*assigned-seat-number*	This indicates the particular seat of the agent within the row. The assigned seat number corresponds with the A, C, D or F letters from the boarding pass, indicating whether the passenger has an aisle or a window seat and the location of this seat, in the left or in the right side of the aisle.
*group-number*	This variable is the boarding group number to which the passenger is assigned. For our purposes, this value varies between 1 and 6 depending on the experiment conducted.
*boarding-index*	The boarding-index is measured for each agent and shows how many passengers have entered the airplane prior to the current agent.
*type-3-seating-interference-state*	This variable shows in every moment whether the agent is involved in a type-3 seat interference situation [35] and contains a true or a false value. More information related to type-3 seat interference is provided in the next section and in [35, 62].
*type-3-seating-interference-time*	This variable represents the time an agent involved in a type-3 seat interference needs to wait in the aisle before taking its seat. The value of this variable is triangularly distributed with a mode of 10 seconds and minimum and maximum values of 9 seconds and 13 seconds respectively consistent with [35, 62].
*aisle-social-distance*	This is the minimum social distance between the agents while moving down the aisle. This aisle social distance can be either 1 m or 2 m.
*time-to-seat*	This variable is the time needed by an agent to take its seat when no additional conditions are met, e.g. storing the luggage, being involved in a seat interference. The value of this variable is equal to 1 tick [35].

The number of ticks required by a passenger for storing carry-on luggage (*luggage-store-ticks*) is given by the following formula [73]:

Tstore=((NbinLarge+0.5NbinSmall+NagentLarge+0.5NagentSmall)*(NagentLarge+0.5NagentSmall)/2)*Trow (1)

Where:

*Tstore* is the time to store the luggage (i.e. *luggage-store-ticks*).

*NbinLarge* is the number of large bags in the overhead bin prior to the agent’s arrival

*NbinSmall* is the number of small bags in the overhead bin prior to the agent’s arrival

*NagentLarge* is the number of large bags carried by the agent (i.e. *luggage-large-no*)

*NagentSmall* is the number of small bags carried by the agent (i.e. *luggage-small-no*)

*Trow* is the time for the agent to walk from one row to the next (when not delayed by another agent in front). It is based on the *default-speed* (and as noted above is generated using the uniform probability distribution)

We assume a continual flow of passengers (except when their progress is impeded by another passenger in front of them). Once the last agent from a group enters the airplane, it is followed by another passenger from the subsequent boarding group (while maintaining the aisle social distance throughout the boarding process). We assume that each passenger boards with its assigned boarding group. To facilitate this, airlines may record the passenger’s boarding group number on the boarding pass (boarding card) and use audio guidance or panel guidance to inform the passengers when their group number should board.

### Scenarios and metrics used for boarding methods evaluation

We test the boarding methods and number of boarding groups using the luggage situations as suggested by [30, 33, 55] and with 1 m and 2 m aisle social distances. The luggage situations feature different frequency percentages of passengers carrying each luggage combination as indicated in Table 2. Among the considered situations, the S7 luggage situation features the case in which none of the passengers carry hand luggage inside the aircraft. This situation has been encountered during the coronavirus pandemic in Italy for a short period of time [74, 75]. The purpose of considering various luggage situations does not advocate or oppose any particular hand luggage policy the airlines may use. These situations have the purpose of investigating a wide range of luggage situations and their resulting impact on the boarding time and passenger health-related performance measures for consideration by the airlines. A fixed number of passengers carrying each number of luggage type is deterministically determined from the luggage situation; the particular passengers carrying each combination of luggage is determined randomly in the stochastic simulation model.

**Table 2:** Luggage situations considered in the simulation experiments.

**Situation**	**Percentages of bags carried by the passengers**				
	0 bag	1 small bag	2 small bags	1 large bag	1 large and 1 small bag
**S1**	10%	10%	0%	10%	70%
**S2**	15%	20%	5%	10%	50%
**S3**	25%	20%	10%	15%	30%
**S4**	35%	25%	10%	15%	15%
**S5**	60%	10%	10%	10%	10%
**S6**	80%	5%	5%	5%	5%
**S7**	100%	0%	0%	0%	0%

The metrics used for evaluating the boarding methods and the number of boarding groups are the same as those presented in [61].

The first performance metric is the average boarding time. The boarding time is the number of seconds between when the first passenger enters the airplane and when all passengers have been seated.

**Fig 12:**
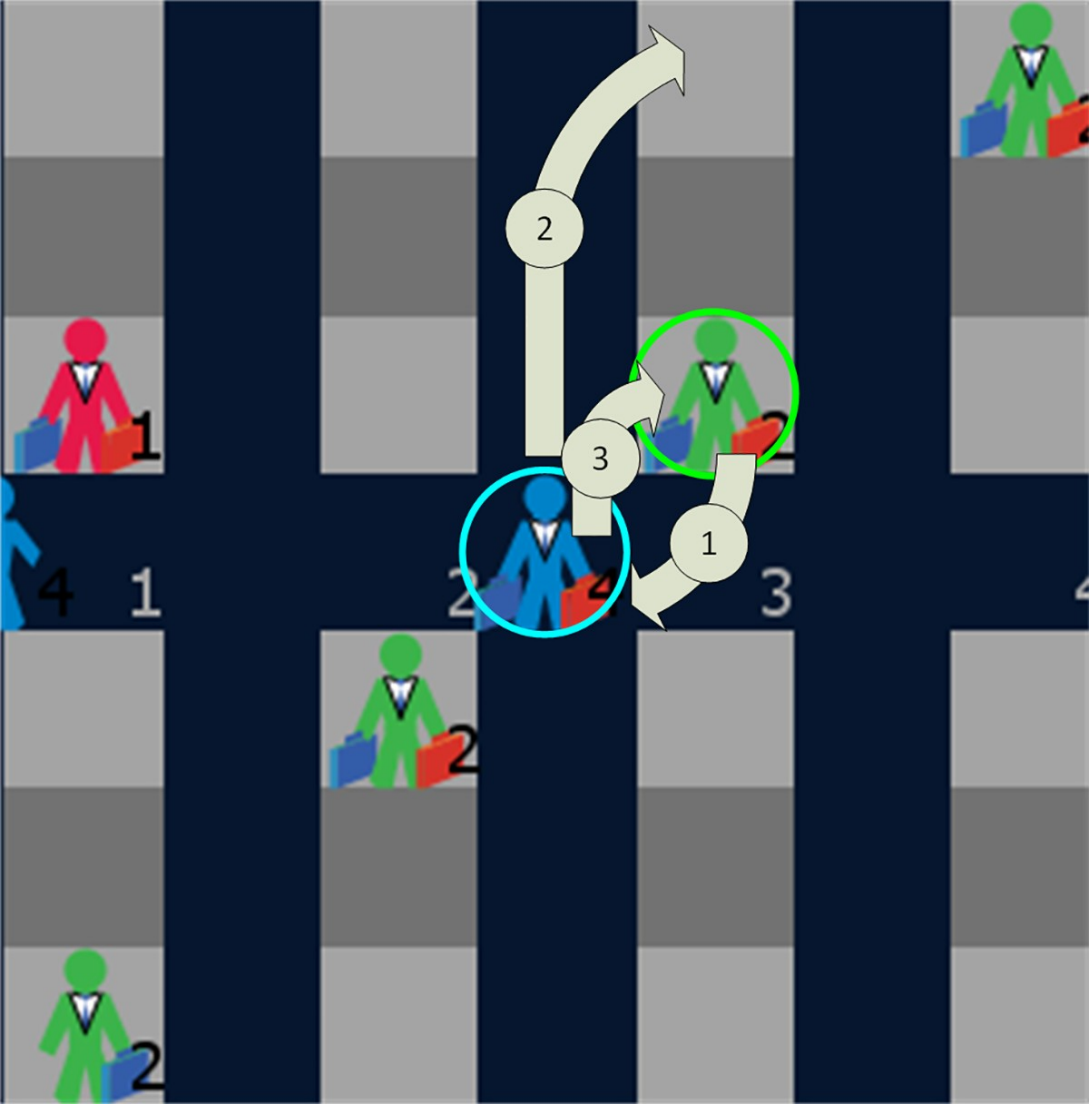
Type-3 seat interference.

The second performance metric is the number of type-3 seat interferences during the boarding. In a type-3 seat interference, a passenger with a window seat arrives at the row of his/her seat (as depicted in blue in Fig 12) after the passenger with an aisle seat (depicted in green in Fig 12) located on the same side of the aisle and in the same row has already occupied his/her assigned seat. As a result, a process with three steps occurs. First, the (green) passenger with the aisle stands to clear the path for the window seat passenger. Second, the (blue) window seat passenger occupies his/her seat. Third, the (green) aisle seat passenger returns to his/her seat.

For the methods used in practice by the airlines, [61] determined that for a similar airplane configuration as the one presented in this paper and with an equal number of passengers and considering 1 m and 2 m aisle social distancing, the average number of type-3 seat interferences can range between 0 and 30. The value of the seat interferences metric is important because the higher the number of type-3 seat interferences, the higher the risk of spreading and inhaling COVID-19 droplets and aerosols by the passengers involved in the seat interference and by nearby passengers.

The third and the fourth performance metrics pertain to seated passengers’ health while later boarding passengers pass them while traversing the aisle towards their seats. The risk to previously seated passengers with aisle seats (aisle seat risk) and window seats (window seat risk) respectively are measured in the total seconds of potential exposure and are calculated respectively as proposed in [61]:

$$AisleSeatRisk=\sum_{p}\sum_{r\leq RowSit_{p}}\left(RowTime_{pr}*\;\sum_{p^{\prime }<p}AisleSeat_{p^{\prime }r}\right)$$ (2)

$$WindowSeatRisk=\sum_{p}\sum_{r\leq RowSit_{p}}\left(RowTime_{pr}*\;\sum_{p^{\prime }<p}WindowSeat_{p^{\prime }r}\right)$$ (3)

where:

*p* = index of passenger advancing towards his/her seat

*r* = row index

*RowSit*_*p*_ = row in which passenger *p* has a seat

*RowTime*_*pr*_ = time that passenger *p* spends in row *r* (this duration begins when passenger *p* begins to enter row *r* and concludes when passenger *p* begins to leave row *r*)

*p’* = index of passenger boarding before passenger *p*

$${AisleSeat}_{p’r}=\left\{ \begin{array}{l} 1\;if\;passenger\;p’\;has\;an\;aisle\;seat\;in\;row\;r\\ 0\;otherwise\; \end{array}\right.$$

$${WindowSeat}_{p’r}=\left\{ \begin{array}{l} 1\;if\;passenger\;p’\;has\;a\;window\;seat\;in\;row\;r\\ 0\;otherwise\; \end{array}\right.$$

As the duration of these two risks increases, the health risk for the previously seated passengers increases commensurately.

## Results

For each experimental condition, we ran 10,000 simulation trials and report the average rounded results in the following. We used the BehaviorSpace tool, provided by NetLogo [68], to run the stochastic simulation experiments.

For the experimental conditions, we varied the aisle social distance from 1 m to 2 m, varied the luggage situations from S1 (high luggage frequencies) to S7 (no luggage), and varied the number of boarding groups from two to six. We use the Reverse Pyramid (RP) boarding method adapted for social distancing as described in the Methods section. With two and three boarding groups, the adaptation is straightforward. With four to six boarding groups, we test both the RP-Steep method and the RP-Spread method. As we vary the experimental conditions, we evaluate the results according to four performance criteria: average boarding time, the number of seat interferences, aisle seat risk, and window seat risk as discussed in each of the following subsections.

### Simulation results for boarding time

The simulation results for the average boarding times with 1 m and 2 m aisle social distances are presented in Tables 3 and 4 respectively. For each given combination (condition) of boarding method, aisle social distance, and a particular number of boarding groups, the average boarding times are higher when more luggage is carried aboard the airplane. For example, the longest boarding times result from the highest luggage scenario S1 and the shortest boarding times result from the no luggage scenario S7. Another unsurprising result is that boarding times increase dramatically when the aisle social distance increases from 1 m to 2 m.

**Table 3:** Average boarding time with 1 m aisle social distance (in seconds).

**Boarding method**	**Number of groups**	**Aisle social distance: 1 m**						
		S1	S2	S3	S4	S5	S6	S7
**RP**	2	952	913	871	833	768	693	548
	3	928	889	847	807	755	677	528
**RP-Spread**	4	906	869	831	793	741	666	518
	5	894	857	823	786	736	666	512
	6	891	856	819	784	734	665	509
**RP-Steep**	4	919	878	838	801	748	671	518
	5	916	874	836	798	746	673	512
	6	915	874	834	798	747	675	509

**Table 4:** Average boarding time with 2 m aisle social distance (in seconds).

**Boarding method**	**Number of groups**	**Aisle social distance: 2 m**						
		S1	S2	S3	S4	S5	S6	S7
**RP**	2	1445	1401	1338	1288	1183	1063	880
	3	1430	1378	1322	1267	1174	1054	861
**RP-Spread**	4	1417	1364	1311	1259	1168	1049	853
	5	1413	1361	1310	1254	1166	1051	848
	6	1414	1365	1310	1256	1170	1054	845
**RP-Steep**	4	1426	1375	1316	1263	1173	1050	853
	5	1425	1374	1318	1263	1175	1058	848
	6	1428	1374	1321	1267	1179	1060	845

**Fig 13:**
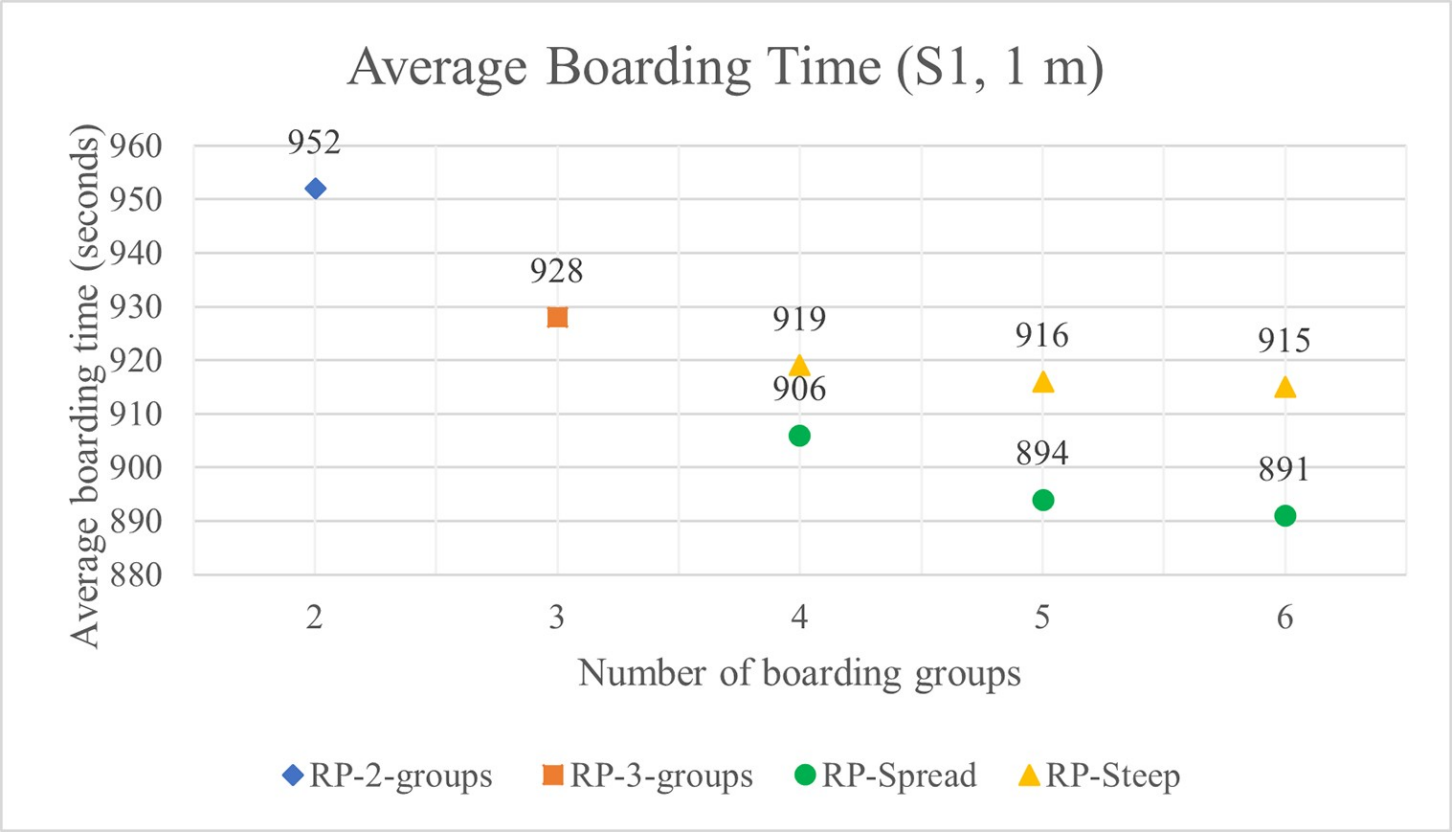
Average boarding time for S1 luggage situation and 1 m aisle social distance.

**Fig 14:**
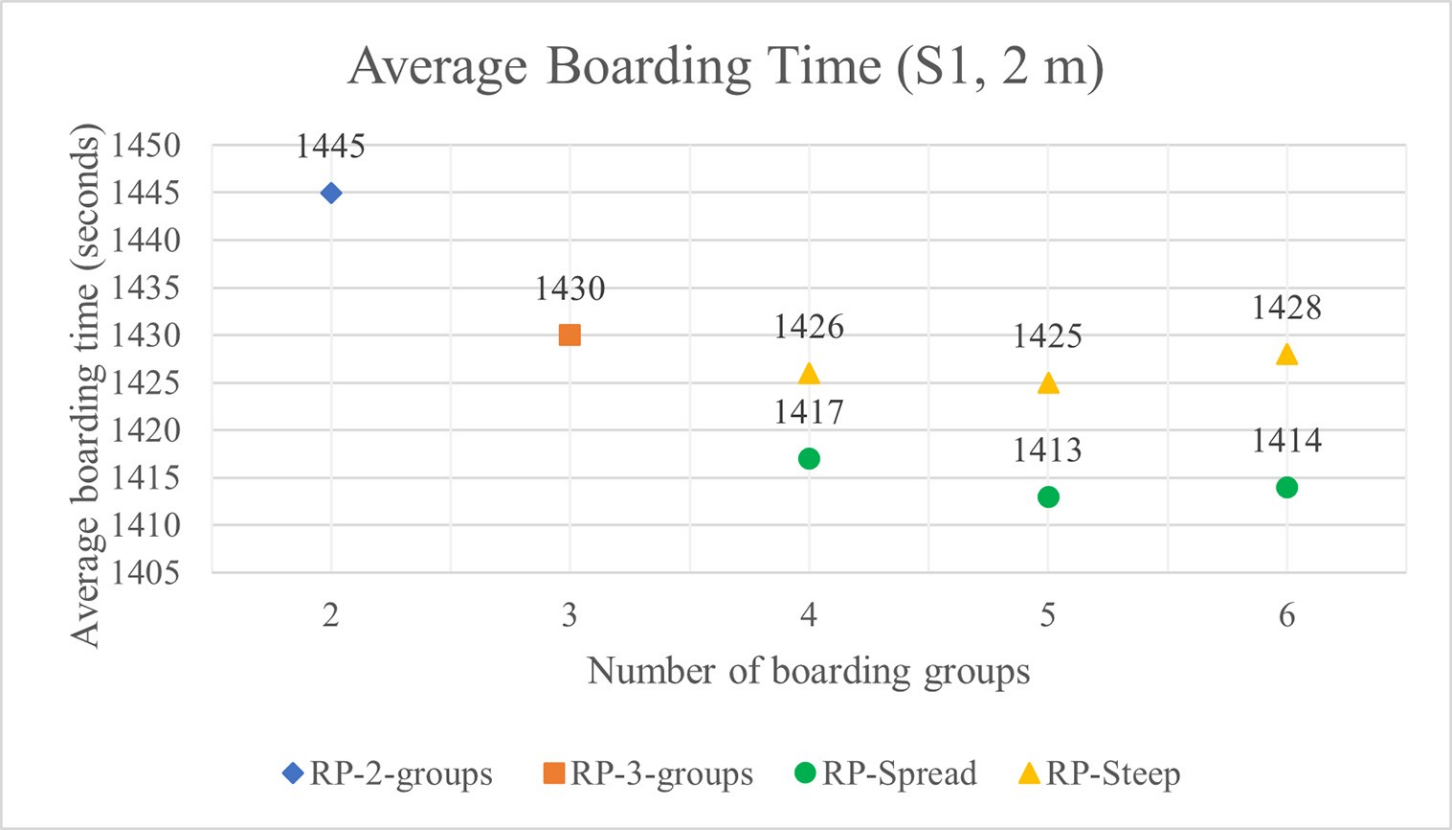
Average boarding time for S1 luggage situation and 2 m aisle social distance.

As the number of boarding groups increases from two to four, average boarding times decrease. As the number of boarding groups increases from four to five, the boarding time advantage of five groups improves slightly when RP-Spread is used except for the relatively low luggage situation S6 where the average difference disappears completely—probably due to the inherent variability of stochastic simulations rather than a significant difference. The boarding method of RP-Spread results in shorter boarding times than RP-Steep. However, the difference in boarding time between the two methods is slight as indicated by the average boarding times shown in Figs 13 and 14 for 1 m and 2 m aisle social distance respectively for the high volume luggage situation S1. Even in this luggage situation, the time difference between the boarding times of RP-Spread and RP-Steep is only 24 seconds and 14 seconds with 6 groups and 1 m and 2 m aisle social distance respectively. With lower volumes of luggage, the differences in boarding times between these two methods shrinks even more to the point of disappearing. Considering all the boarding time results, we conclude that increasing the number of boarding groups to four decreases average boarding times—particularly for the higher volume luggage situations—and after that, any reduction in boarding times from increasing the number of groups beyond four is small to non-existent. We further conclude that RP-Spread has slight boarding time advantages over RP-Steep and with meaningful differences only for the higher volume luggage situations.

### Simulation results for type-3 seat interferences

All of the adapted Reverse Pyramid methods board the window seat passengers of each row in an earlier boarding group than the row’s aisle seat passengers. Consequently, it is not surprising that there are zero type-3 seat interferences for all experimental conditions tested. This is a strength of Reverse Pyramid.

### Simulation results for aisle seat risk duration

**Fig 15:**
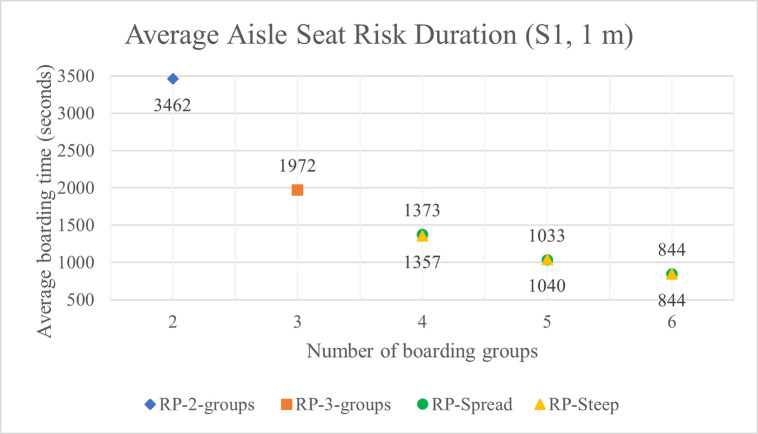
Average aisle seat risk duration for S1 luggage situation and 1 m aisle social distance.

**Fig 16:**
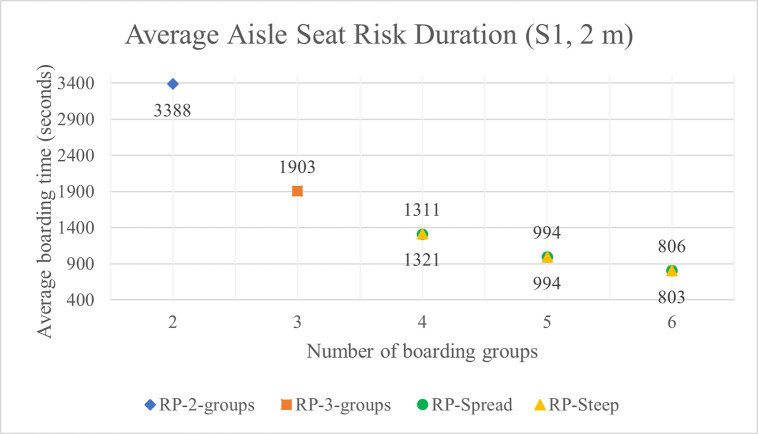
Average aisle seat risk duration for S1 luggage situation and 2 m aisle social distance.

The average aisle seat risk durations with 1 m and 2 m aisle social distance are shown in Tables 5 and 6 have respectively. For the high luggage volume situation S1, the aisle seat risk is shown as a function of the number of boarding groups in Fig 15 and in Fig 16 with 1 m and 2 m aisle social distance respectively.

**Table 5:** Average aisle seat risk duration with 1 m aisle social distance (in seconds).

**Boarding method**	**Number of groups**	**Aisle social distance: 1 m**						
		S1	S2	S3	S4	S5	S6	S7
**RP**	2	3462	3252	3118	3023	2759	2534	2087
	3	1972	1870	1760	1639	1519	1363	1176
**RP-Spread**	4	1373	1262	1180	1088	990	897	774
	5	1033	962	881	814	729	647	571
	6	844	774	691	640	558	508	441
**RP-Steep**	4	1357	1270	1194	1104	990	891	779
	5	1040	953	881	815	729	654	569
	6	844	772	699	632	569	506	438

**Table 6:** Average aisle seat risk duration with 2 m aisle social distance (in seconds).

**Boarding method**	**Number of groups**	**Aisle social distance: 2 m**						
		S1	S2	S3	S4	S5	S6	S7
**RP**	2	3388	3254	3056	2954	2711	2486	2126
	3	1903	1797	1717	1611	1455	1313	1163
**RP-Spread**	4	1311	1234	1147	1054	961	869	775
	5	994	920	853	782	710	639	568
	6	806	749	678	613	557	500	445
**RP-Steep**	4	1321	1229	1143	1066	964	870	778
	5	994	930	860	789	717	645	565
	6	803	737	678	620	550	498	444

In all conditions, when the number of boarding groups increases, the aisle seat risk decreases significantly. As the number of groups increases from 2 to 6, the reduction in aisle seat risk from the previous number of group’s risk averages about 44%, 33%, 26%, and 21% for groups 3 to 6 respectively, thus indicating diminishing (but still significant) value in aisle seat risk reduction. The variability in these percentages (only about +/- 2%) is consistent across all experimental conditions of aisle social distance, luggage situation, and whether the RP-Spread or RP-Steep method is used.

There is no pattern in either RP-Spread or RP-Steep providing lower aisle seat risk than the other boarding method. Any differences in aisle seat risk between the two boarding methods likely stems from the variability inherent in the uncertainties of stochastic simulation.

As the volume of luggage carried aboard the airplane decreases, the aisle seat risk duration decreases significantly. For instance, with four boarding groups, aisle social distance of 1 m, and RP-Spread, the no luggage situation S7 (and medium luggage situation S4) has 44% (and 21%) less aisle seat risk than the high volume luggage situation S1. This suggests that airlines that discourage or prohibit passengers from carrying (much) luggage aboard the airplane would keep them safer.

**Fig 17:**
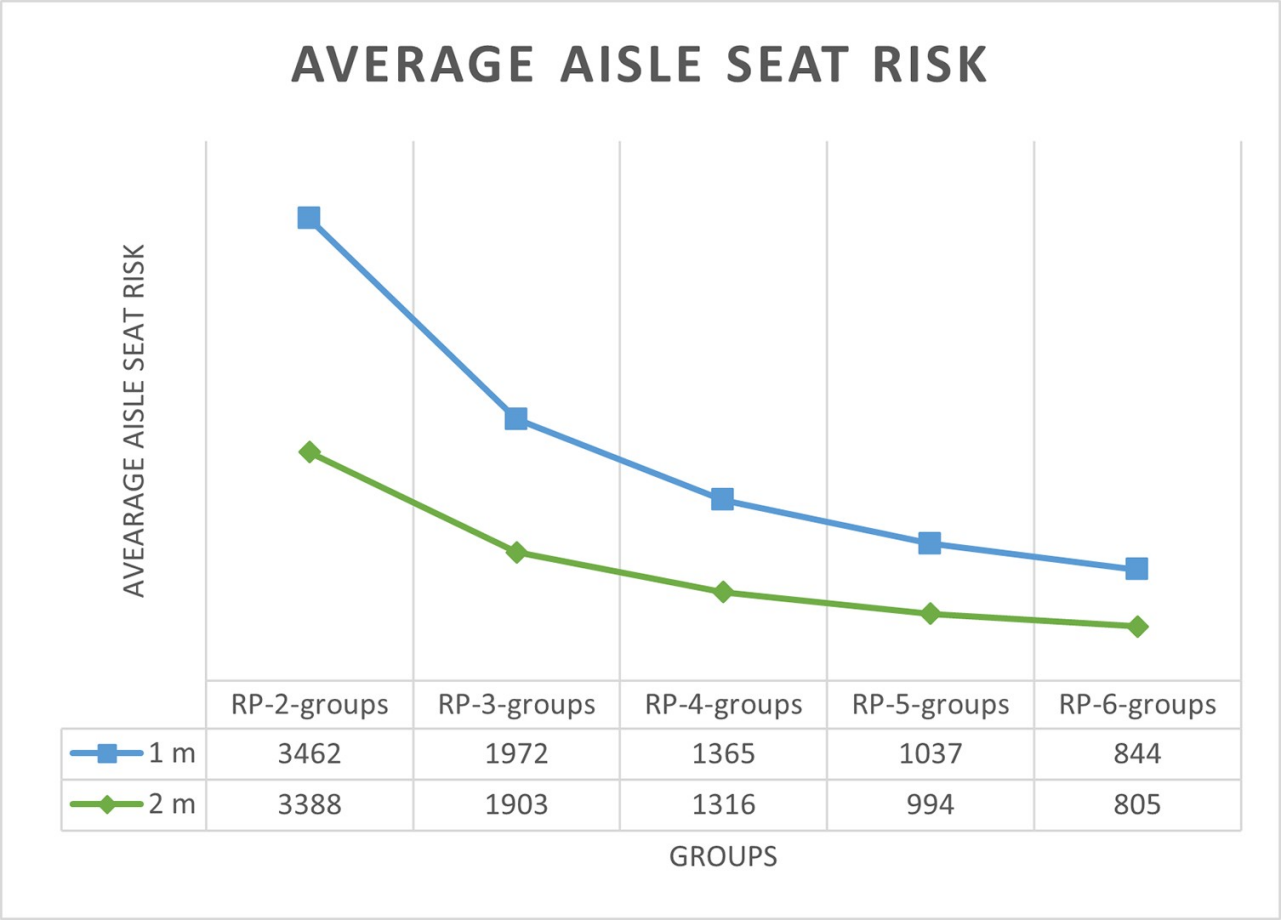
Average aisle seat risk duration for S1 luggage situation for 1 m and 2 m aisle social distances.

Fig 17 for the S1 luggage situation illustrates the favorable impact on aisle seat risk when the aisle social distance doubles from 1 m to 2 m. In this figure, the values of aisle seat risk for the number of groups of 4, 5, and 6 are the average of their values for RP-Spread and RP-Steep for the same number of boarding groups. The reduction in aisle seat risk from doubling the aisle social distance diminishes in absolute terms as the number of boarding groups increases but remains significant even with 6 boarding groups.

### Simulation results for window seat risk duration

**Fig 18:**
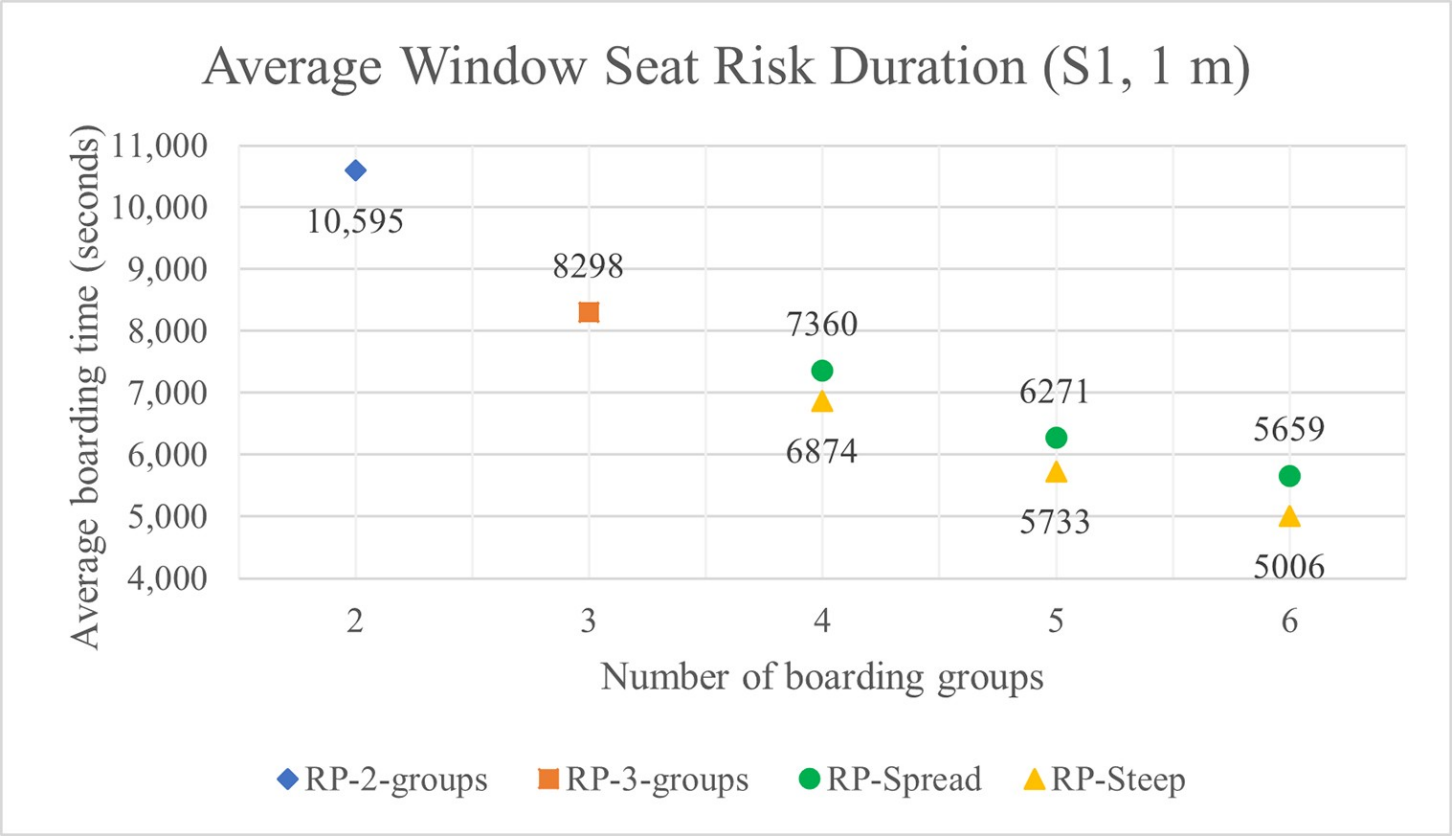
Average window seat risk duration for S1 luggage situation and 1 m aisle social distancing.

**Fig 19:**
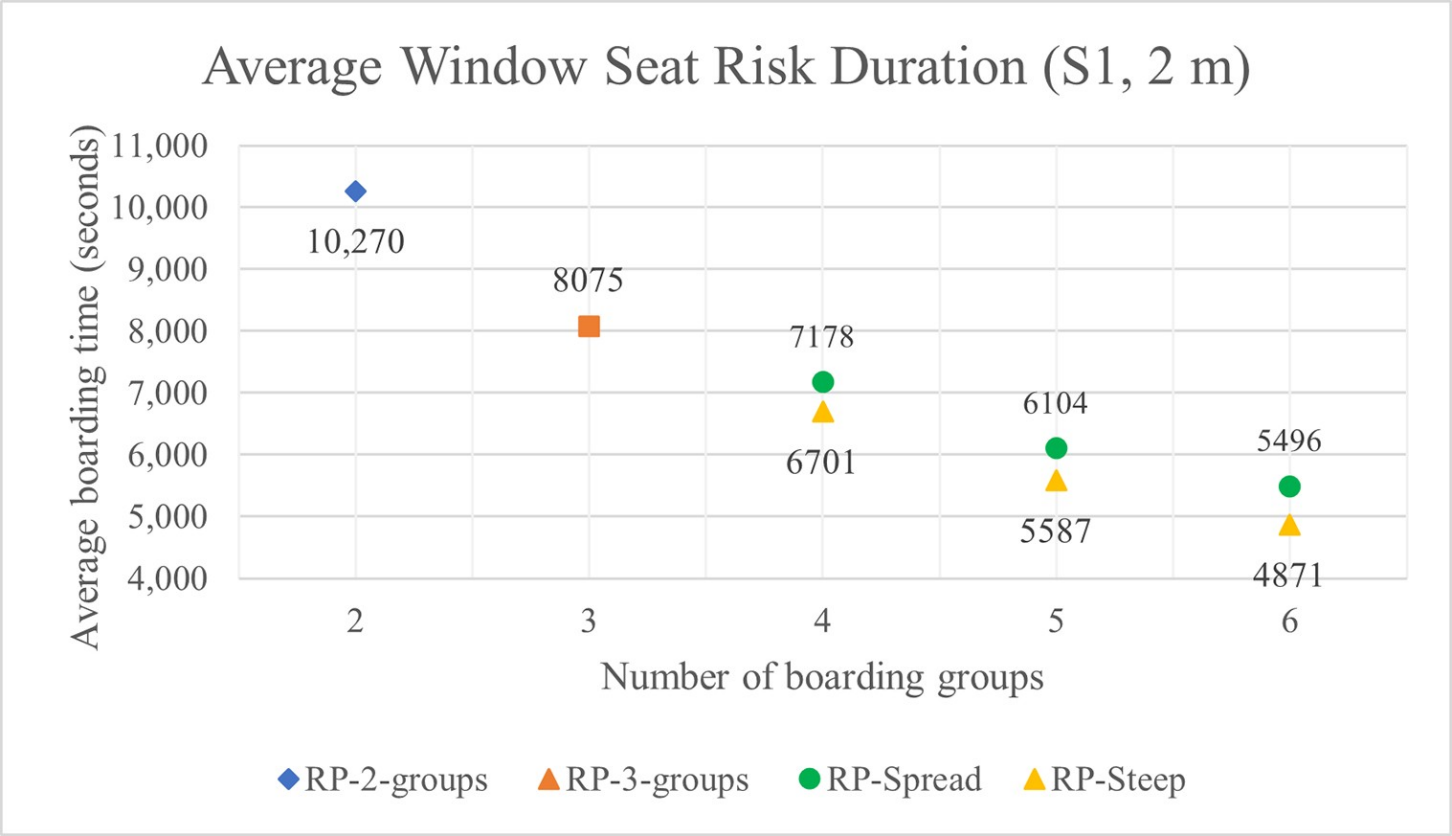
Average window seat risk duration for S1 luggage situation and 2 m aisle social distancing.

The average window seat risk durations with 1 m and 2 m aisle social distances are shown in Tables **7** and **8** and for the S1 luggage situation in Figs **18** and **19**. As indicated in the tables and figures, many of the key relationships between window seat risk durations and other factors are the same as those between aisle seat risk durations and those other factors. For instance, as with aisle seat risk, the window seat risk decreases when the number of boarding groups increases, the volume of luggage carried aboard the airplane decreases, and the aisle social distance doubles.

**Table 7:** Average window seat risk duration with 1 m aisle social distancing (in seconds).

**Boarding method**	**Number of groups**	**Aisle social distancing: 1 m**						
		S1	S2	S3	S4	S5	S6	S7
**RP**	2	10,595	10,152	9658	9203	8472	7668	6460
	3	8298	7897	7514	7093	6519	5869	4991
**RP-Spread**	4	7360	6992	6602	6226	5714	5106	4362
	5	6271	5903	5552	5179	4733	4252	3602
	6	5659	5283	4925	4594	4170	3770	3186
**RP-Steep**	4	6874	6483	6129	5772	5261	4747	4089
	5	5733	5398	5038	4712	4303	3853	3326
	6	5006	4661	4313	4028	3658	3276	2849

**Table 8:** Average window seat risk duration with 2 m aisle social distance (in seconds).

**Boarding method**	**Number of groups**	**Aisle social distance: 2 m**						
		S1	S2	S3	S4	S5	S6	S7
**RP**	2	10,270	9925	9383	8988	8215	7444	6434
	3	8075	7696	7303	6919	6344	5727	5006
**RP-Spread**	4	7178	6796	6425	6086	5559	5003	4362
	5	6104	5763	5417	5032	4587	4140	3627
	6	5496	5164	4821	4477	4073	3669	3190
**RP-Steep**	4	6701	6352	5964	5645	5146	4634	4083
	5	5587	5251	4927	4598	4171	3769	3336
	6	4871	4546	4240	3927	3571	3205	2846

Unlike with aisle seat risk, we observe that window seat risk is consistently reduced when RP-Steep is used instead of RP-Spread. RP-Spread results in an average window seat risk reduction of about 7%, 9%, and 12% when the number of boarding groups are 4, 5, and 6 respectively—a relationship that is consistent (+/- 1%) across all luggage situations and with both 1 m and 2 m aisle social distance.

## Discussion

Based on the simulation results for each method in the considered luggage situations, we observe that increasing the number of boarding groups from two to four decreases boarding times—particularly for the higher volume luggage situations—and that any reduction in boarding times from increasing the number of groups beyond four is small to non-existent. The RP-Spread method has slightly shorter boarding times than RP-Steep, particularly with the higher volume luggage situations. On the other hand, the RP-Steep method has lower window seat risk than the RP-Spread method for the same number of groups.

All of the adapted Reverse Pyramid methods are designed to have zero type-3 seat interferences, and the simulation experiments confirm this.

As the number of boarding groups increases, the aisle and window seat risks both decrease. The aisle and window seat risk durations also decrease when the volume of luggage carried aboard the airplane decreases. Reducing carry-on luggage reduces boarding times.

Doubling the aisle social distance from 1 m to 2 m increases the average boarding time and decreases both aisle and window seat risks.

## Conclusions

In the present paper, we adapt the Reverse Pyramid method for social distancing conditions of the airplane’s middle seats being unoccupied and boarding passengers maintaining a minimum aisle social distance between them. Our adaptations adjust for a varying number of boarding groups (from two to six) and are designed to minimize the health risks caused by seat interferences (which are zero with our methods) and apply three design principles to minimize the health risk to passengers seated adjacent to the aisle from later-boarding (potentially infectious) passengers walking in the aisle near them.

We use stochastic simulation and agent-based modelling to show the resulting impact on four performance metrics while varying the number of boarding groups, the volume of luggage carried, and the aisle social distance. Increasing the number of boarding groups from two to six reduces boarding time up to four groups but continues to reduce infection risk up to six groups. One adaptation of the Reverse Pyramid method (RP-Spread) provides slightly faster boarding times than the other (RP-Steep), when luggage volumes are high, while RP-Steep results in less risk to window seat passengers from later-boarding passengers walking by their seat’s row.

Airlines may want to encourage passengers to carry fewer luggage aboard the airplane. Less luggage would reduce the time to board the airplane and also reduce health risks to passengers. Increasing the aisle social distance from 1 m to 2 m lengthens boarding times but results in lower risks of infection transmission for both walking and previously seated passengers.

In summary, the paper provides effective adaptations of the Reverse Pyramid boarding method for social distancing, and provides insight to airline management as they consider the health and boarding time impacts of their policy decisions on: the number of boarding groups, carry-on luggage restrictions, and the aisle social distance they suggest to their passengers.

Future research may investigate how boarding methods can be tightly integrated with the modeling of infectious disease spread as medical specialists continue to learn about the transmission of SARS-CoV2. Additional research may examine the implication of the number of boarding groups changing when apron buses are used instead of jet bridges. Another research opportunity is analyzing the sensitivity of boarding methods to varying occupancy levels within a flight when considering the social distancing norms imposed by the coronavirus outbreak. Also, the use of the work with different seat assignment policies may be investigated (e.g. leaving every other row unoccupied).

The paper is accompanied by a series of videos made for S1 luggage situation, for all the considered methods, for both 1 m and 2 m aisle social distancing (S1–S16 Figs). The paper is further accompanied by the input files of the simulation experiments (S15–S28 Files) and the output files (experimental results, S1–S14 Files) of those simulations, that in turn, are used in creating the resulting tables and figures that are based on the numerical experiments. The videos, input, and output files can be accessed at the following link: https://github.com/liviucotfas/PlosOne-airplane-boarding-adapting-rp-covid19

## Supporting information

S1 Fig Video recording of RP with 2 boarding groups and 1 m social distancing. (GIF)

S2 Fig Video recording of RP with 3 boarding groups and 1 m social distancing. (GIF)

S3 Fig Video recording of RP-Spread with 4 boarding groups and 1 m social distancing. (GIF)

S4 Fig Video recording of RP-Spread with 5 boarding groups and 1 m social distancing. (GIF)

S5 Fig Video recording of RP-Spread with 6 boarding groups and 1 m social distancing. (GIF)

S6 Fig Video recording of RP-Steep with 4 boarding groups and 1 m social distancing. (GIF)

S7 Fig Video recording of RP-Steep with 5 boarding groups and 1 m social distancing. (GIF)

S8 Fig Video recording of RP-Steep with 6 boarding groups and 1 m social distancing. (GIF)

S9 Fig Video recording of RP with 2 boarding groups and 2 m social distancing. (GIF)

S10 Fig Video recording of RP with 3 boarding groups and 2 m social distancing. (GIF)

S11 Fig Video recording of RP-Spread with 4 boarding groups and 2 m social distancing. (GIF)

S12 Fig Video recording of RP-Spread with 5 boarding groups and 2 m social distancing. (GIF)

S13 Fig Video recording of RP-Spread with 6 boarding groups and 2 m social distancing. (GIF)

S14 Fig Video recording of RP-Steep with 4 boarding groups and 2 m social distancing. (GIF)

S15 Fig Video recording of RP-Steep with 5 boarding groups and 2 m social distancing. (GIF)

S16 Fig Video recording of RP-Steep with 6 boarding groups and 2 m social distancing. (GIF)

S1 File Experimental results for S1 luggage situation and 1 m social distancing. (CSV)

S2 File Experimental results for S2 luggage situation and 1 m social distancing. (CSV)

S3 File Experimental results for S3 luggage situation and 1 m social distancing. (CSV)

S4 File Experimental results for S4 luggage situation and 1 m social distancing. (CSV)

S5 File Experimental results for S5 luggage situation and 1 m social distancing. (CSV)

S6 File Experimental results for S6 luggage situation and 1 m social distancing. (CSV)

S7 File Experimental results for S7 luggage situation and 1 m social distancing. (CSV)

S8 File Experimental results for S1 luggage situation and 2 m social distancing. (CSV)

S9 File Experimental results for S2 luggage situation and 2 m social distancing. (CSV)

S10 File Experimental results for S3 luggage situation and 2 m social distancing. (CSV)

S11 File Experimental results for S4 luggage situation and 2 m social distancing. (CSV)

S12 File Experimental results for S5 luggage situation and 2 m social distancing. (CSV)

S13 File Experimental results for S6 luggage situation and 2 m social distancing. (CSV)

S14 File Experimental results for S7 luggage situation and 2 m social distancing. (CSV)

S15 File Experiment configuration for S1 luggage situation and 1 m social distancing. (XML)

S16 File Experiment configuration for S2 luggage situation and 1 m social distancing. (XML)

S17 File Experiment configuration for S3 luggage situation and 1 m social distancing. (XML)

S18 File Experiment configuration for S4 luggage situation and 1 m social distancing. (XML)

S19 File Experiment configuration for S5 luggage situation and 1 m social distancing. (XML)

S20 File Experiment configuration for S6 luggage situation and 1 m social distancing. (XML)

S21 File Experiment configuration for S7 luggage situation and 1 m social distancing. (XML)

S22 File Experiment configuration for S1 luggage situation and 2 m social distancing. (XML)

S23 File Experiment configuration for S2 luggage situation and 2 m social distancing. (XML)

S24 File Experiment configuration for S3 luggage situation and 2 m social distancing. (XML)

S25 File Experiment configuration for S4 luggage situation and 2 m social distancing. (XML)

S26 File Experiment configuration for S5 luggage situation and 2 m social distancing. (XML)

S27 File Experiment configuration for S6 luggage situation and 2 m social distancing. (XML)

S28 File Experiment configuration for S7 luggage situation and 2 m social distancing. (XML)
