# Supplementary figures and images for: Adapting the reverse pyramid airplane boarding method for social distancing in times of COVID-19
Source: PLoS One. 2020 Nov 4;15(11):e0242131. doi: 10.1371/journal.pone.0242131 (PMC7641653; doi:10.1371/journal.pone.0242131)

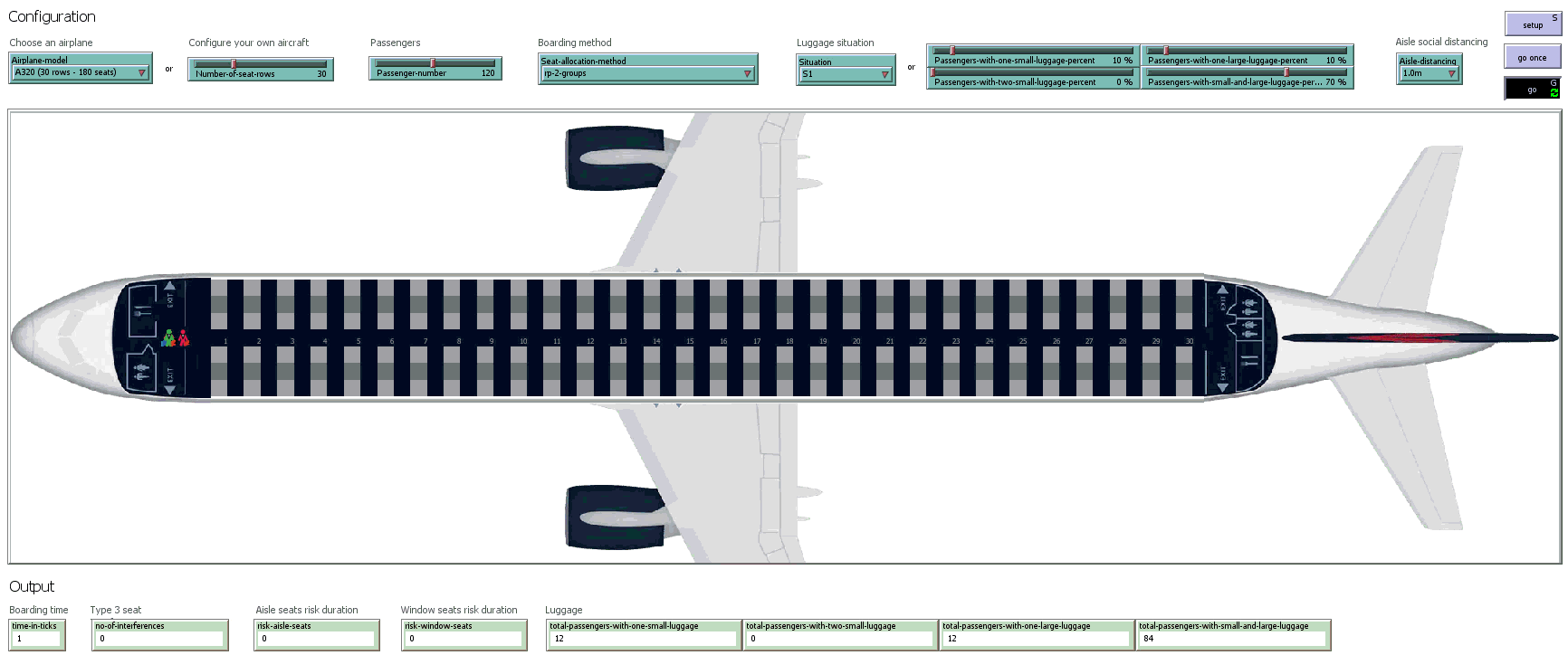

Supplement: S1 Fig — (GIF) [file pone.0242131.s001.gif]

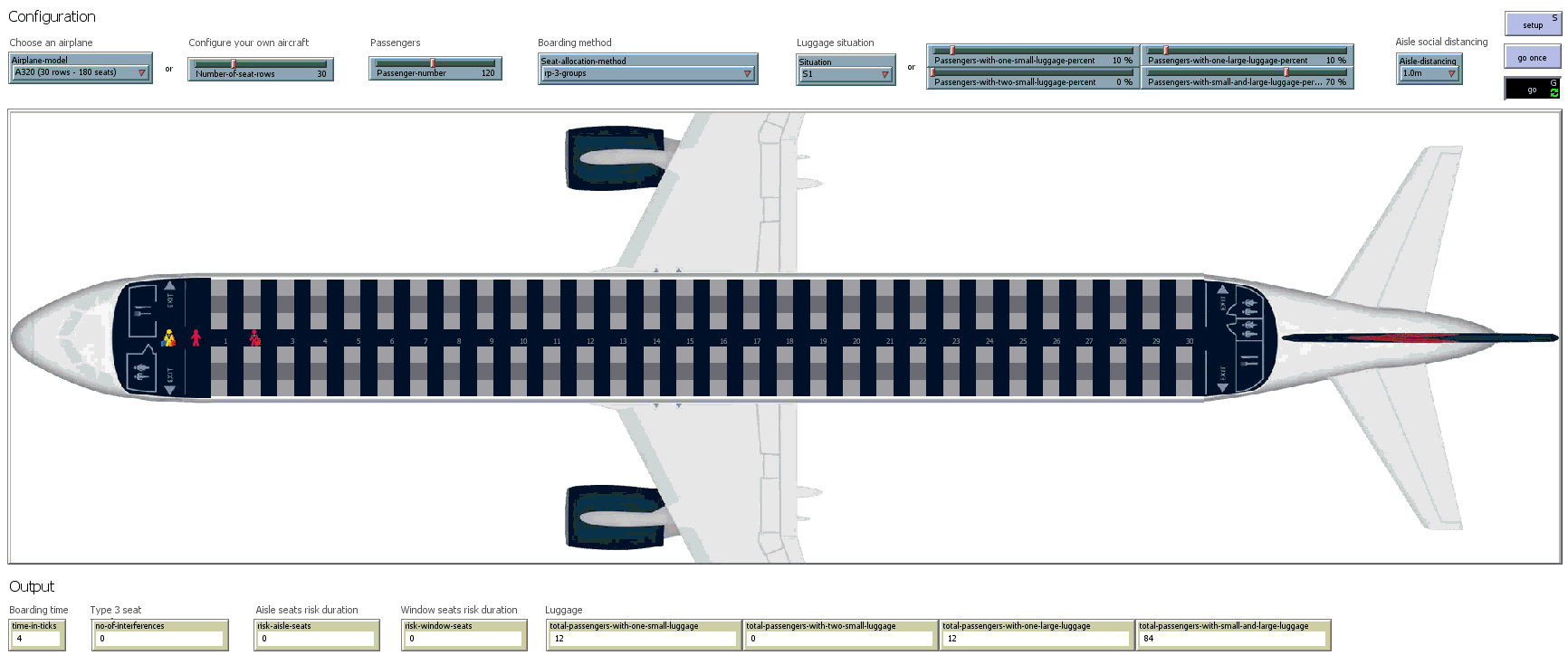

Supplement: S2 Fig — (GIF) [file pone.0242131.s002.gif]

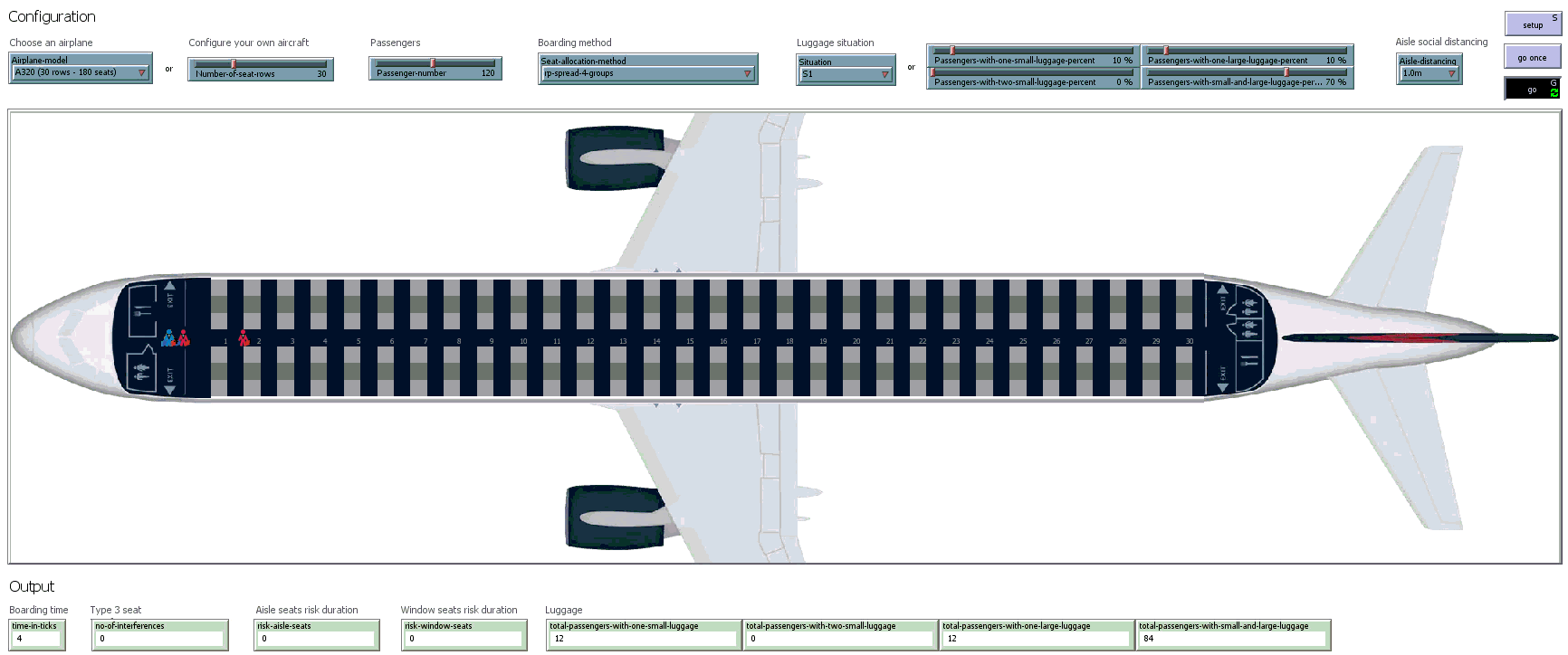

Supplement: S3 Fig — (GIF) [file pone.0242131.s003.gif]

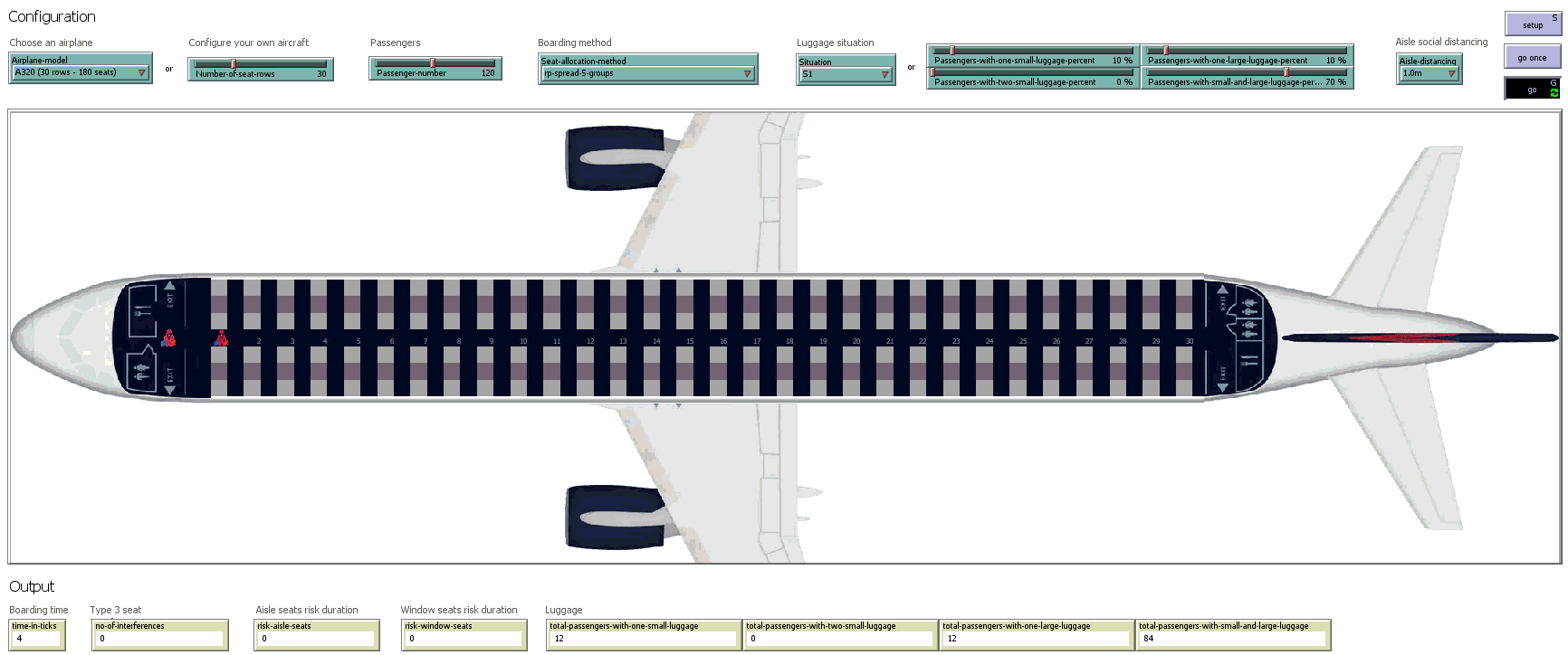

Supplement: S4 Fig — (GIF) [file pone.0242131.s004.gif]

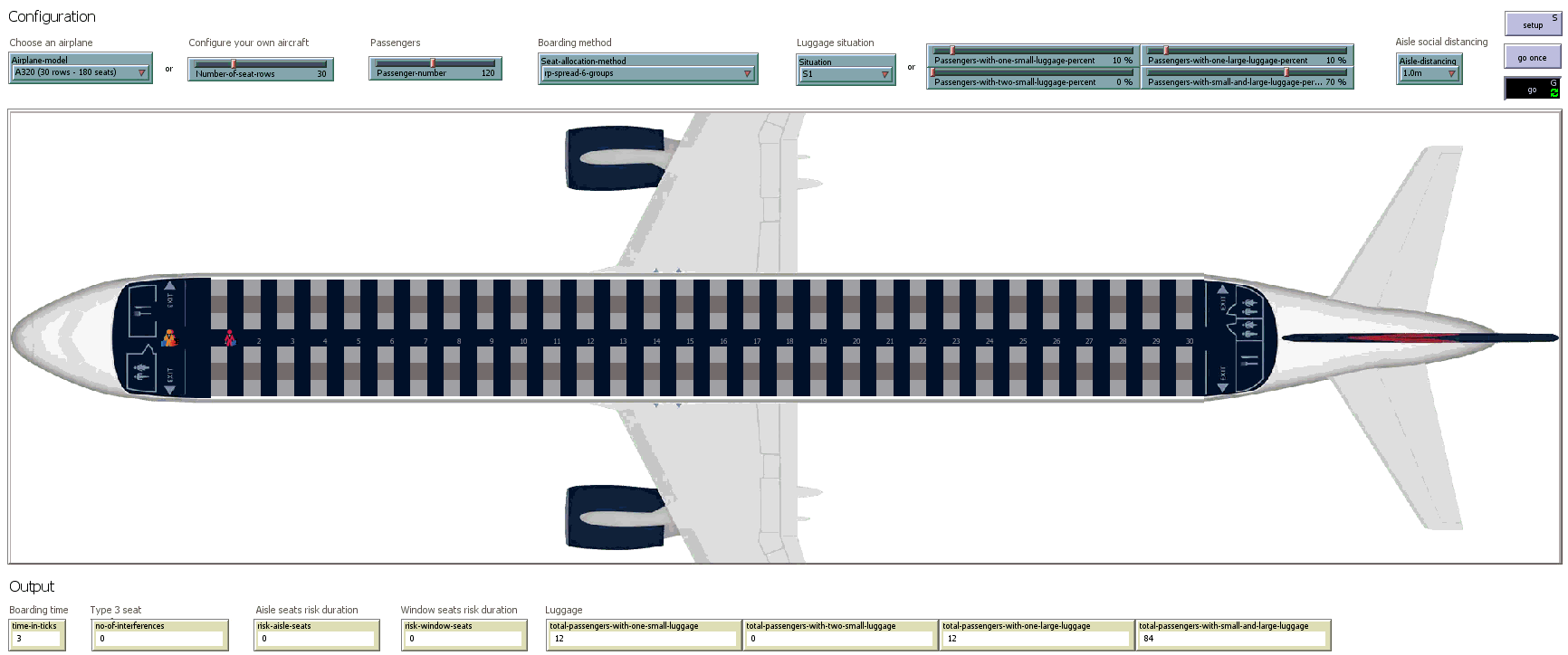

Supplement: S5 Fig — (GIF) [file pone.0242131.s005.gif]

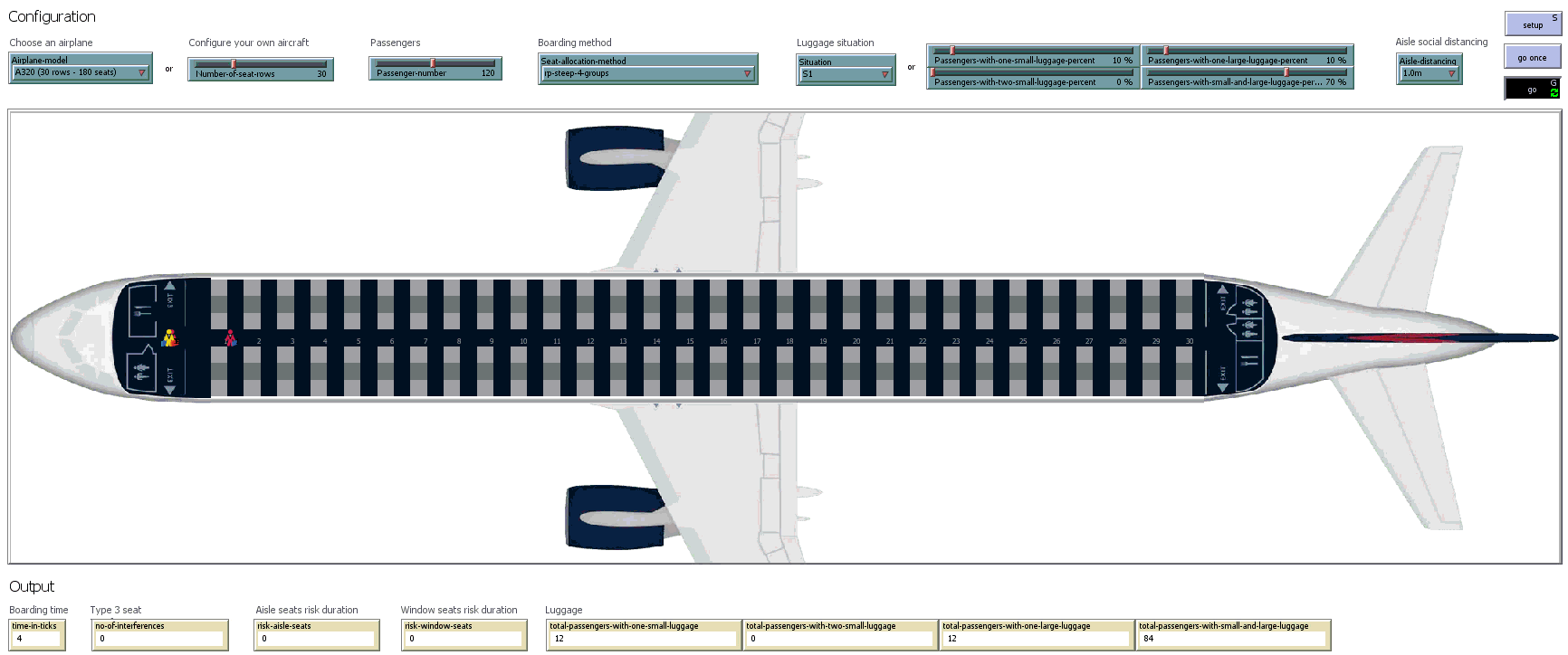

Supplement: S6 Fig — (GIF) [file pone.0242131.s006.gif]

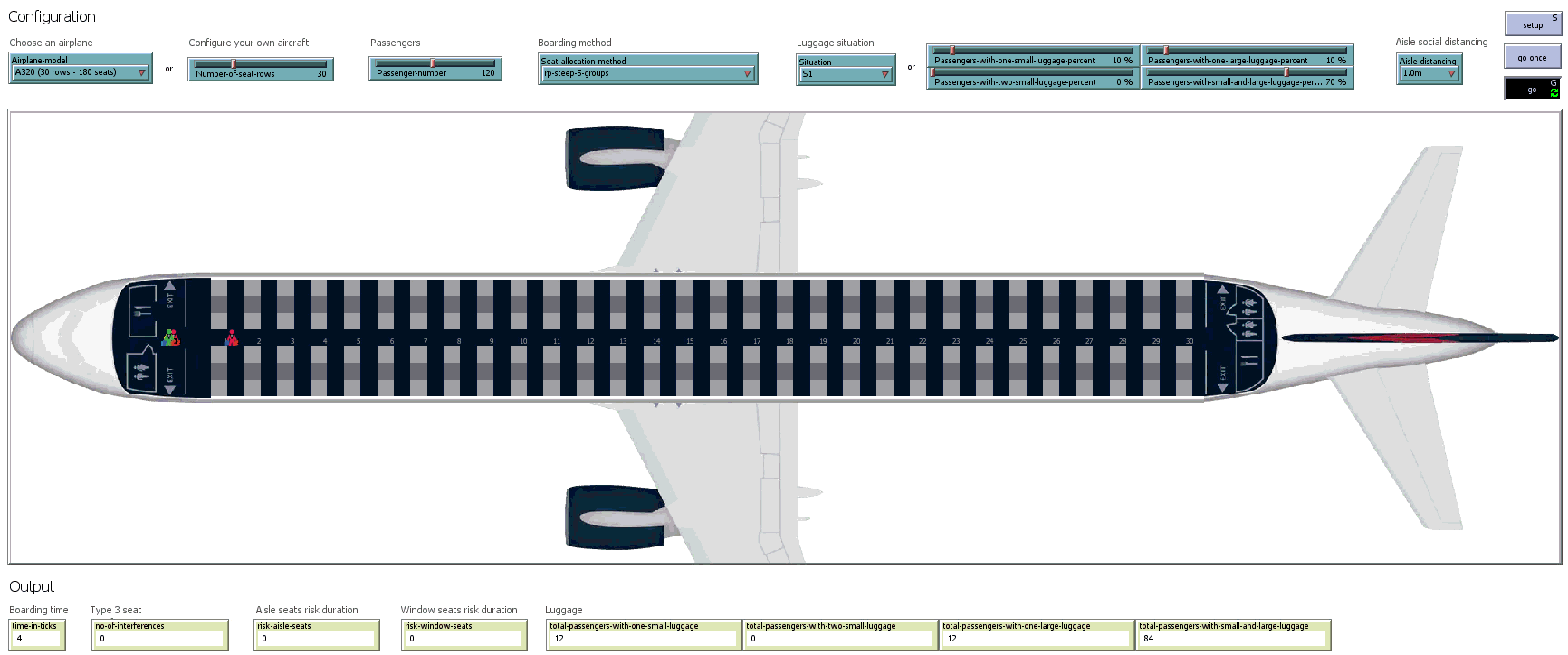

Supplement: S7 Fig — (GIF) [file pone.0242131.s007.gif]

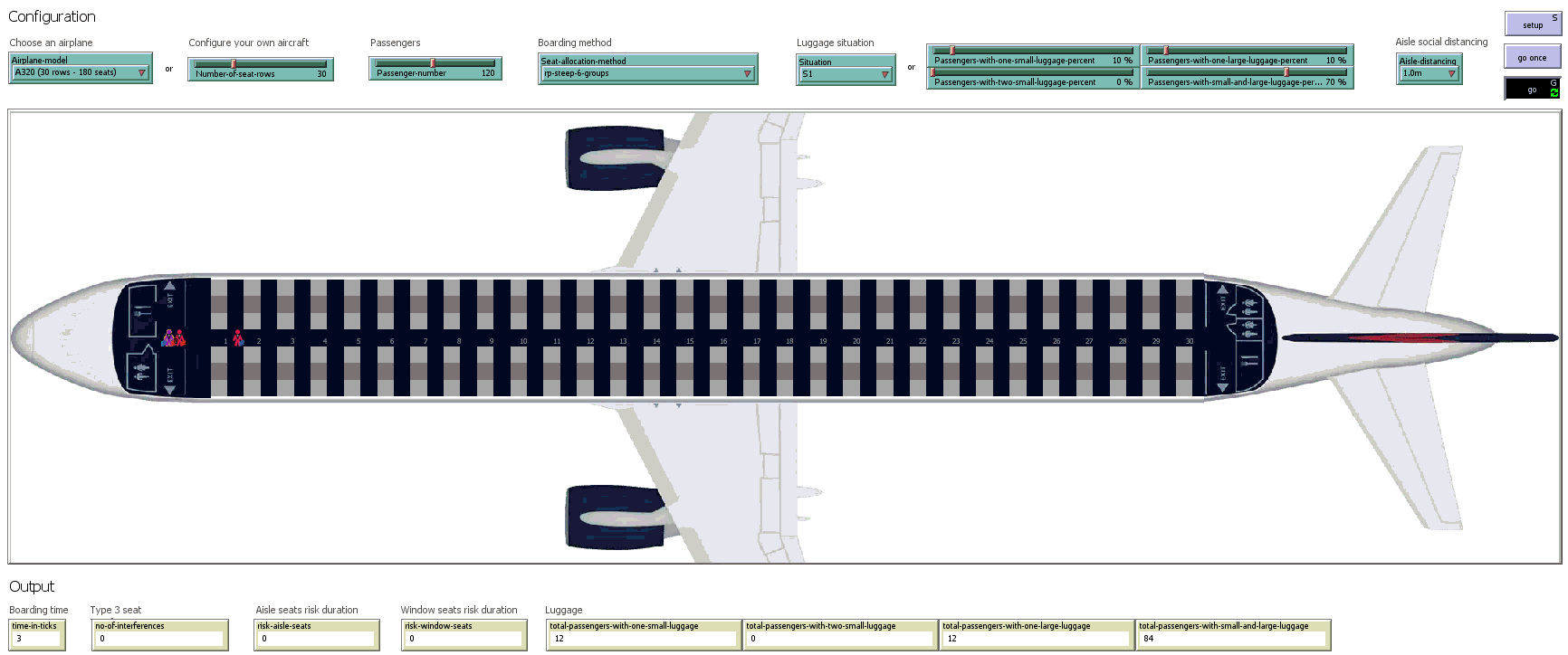

Supplement: S8 Fig — (GIF) [file pone.0242131.s008.gif]

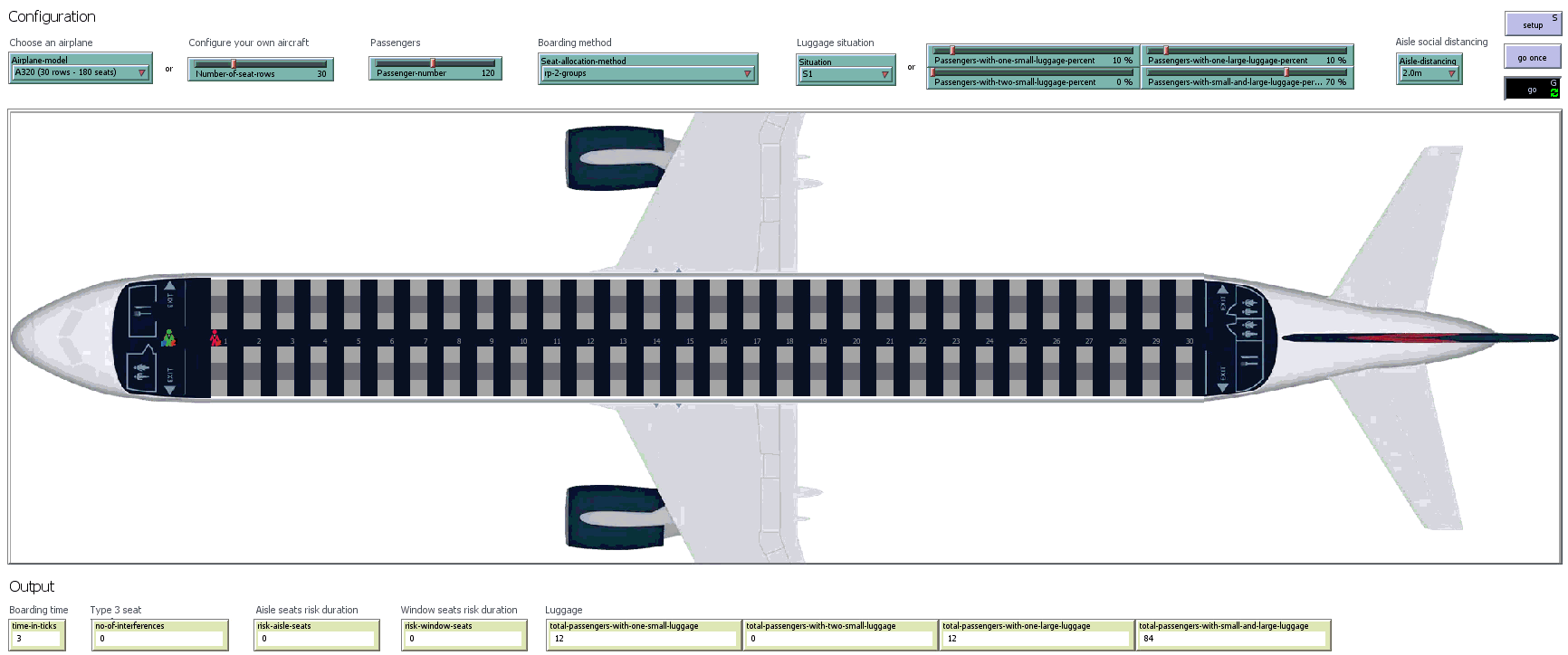

Supplement: S9 Fig — (GIF) [file pone.0242131.s009.gif]

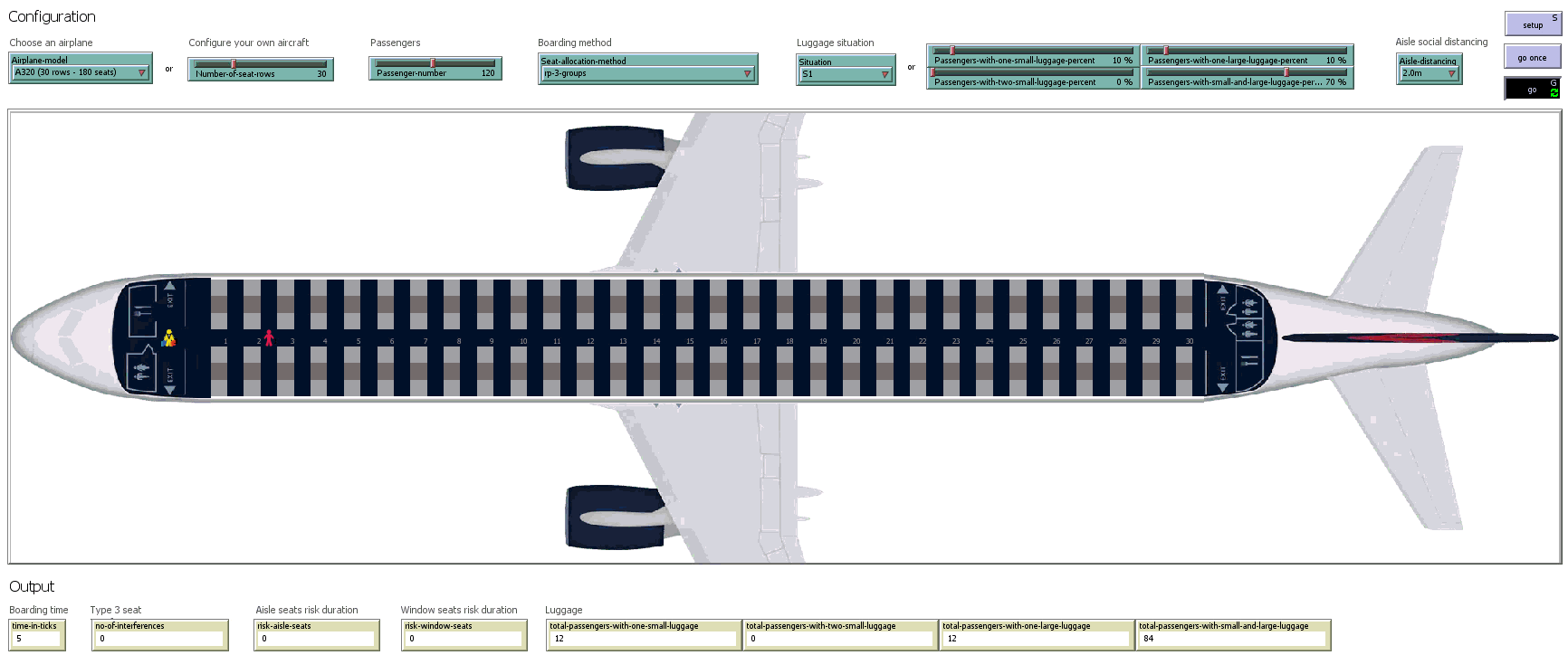

Supplement: S10 Fig — (GIF) [file pone.0242131.s010.gif]

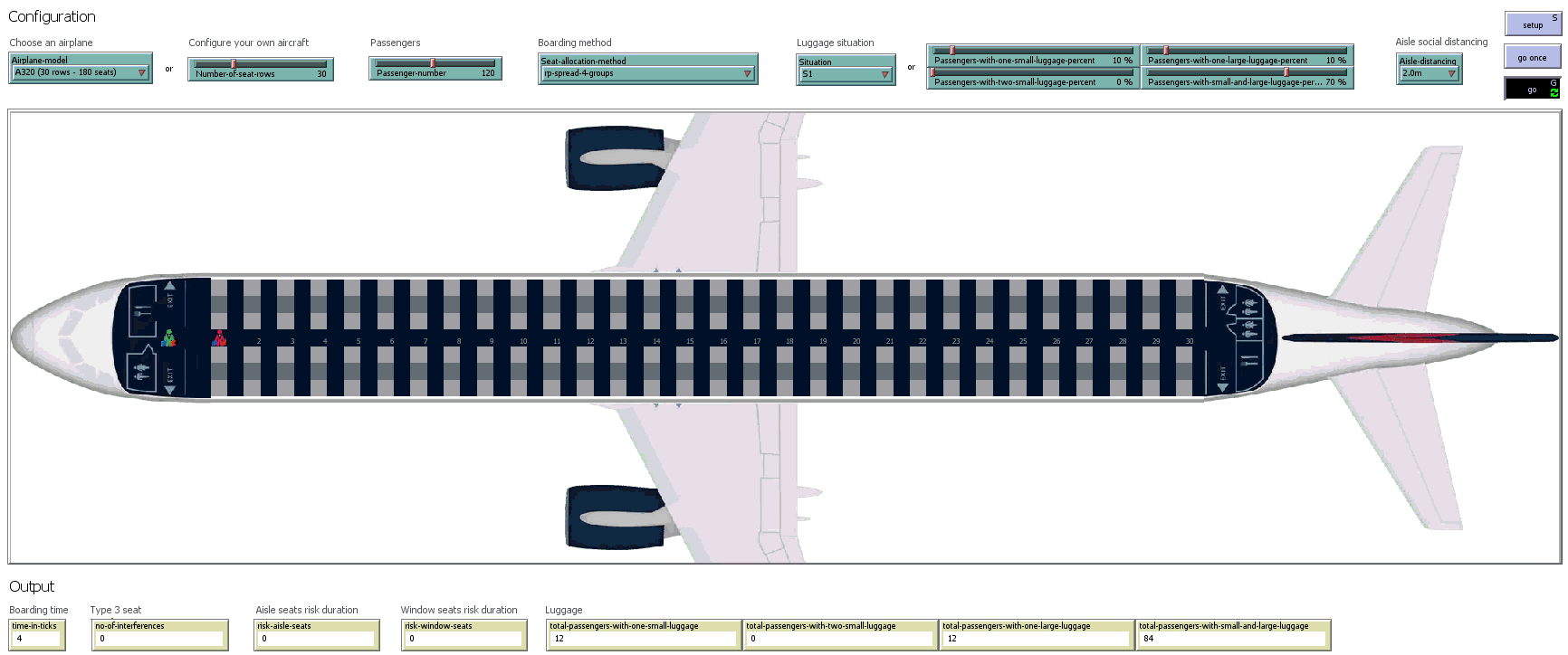

Supplement: S11 Fig — (GIF) [file pone.0242131.s011.gif]

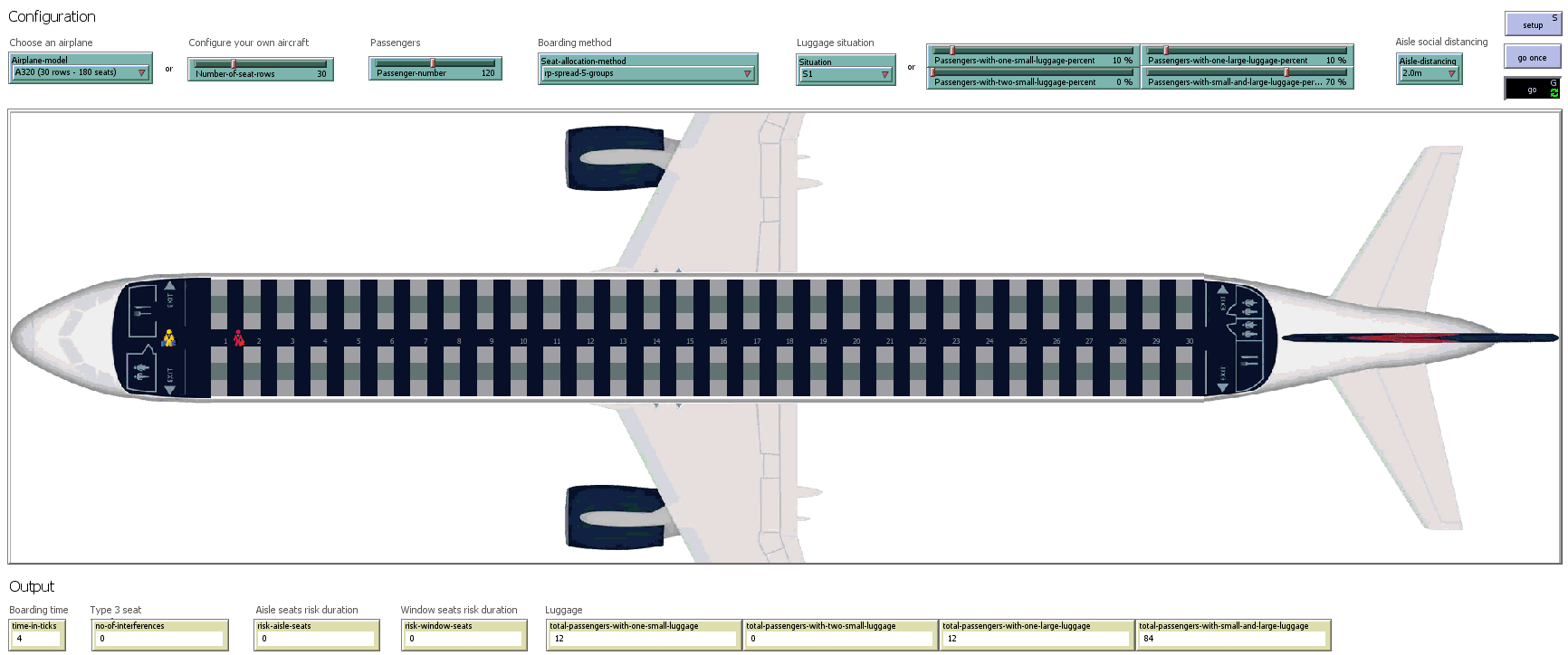

Supplement: S12 Fig — (GIF) [file pone.0242131.s012.gif]

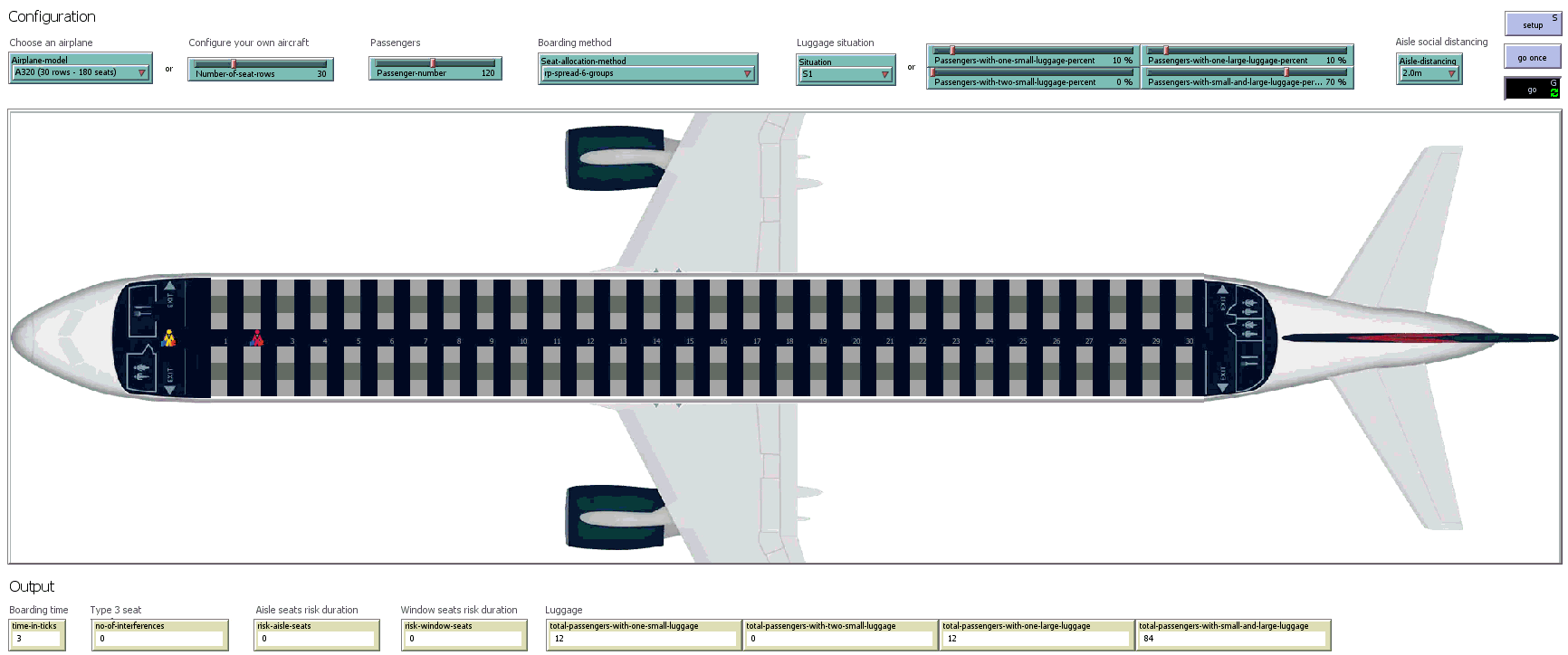

Supplement: S13 Fig — (GIF) [file pone.0242131.s013.gif]

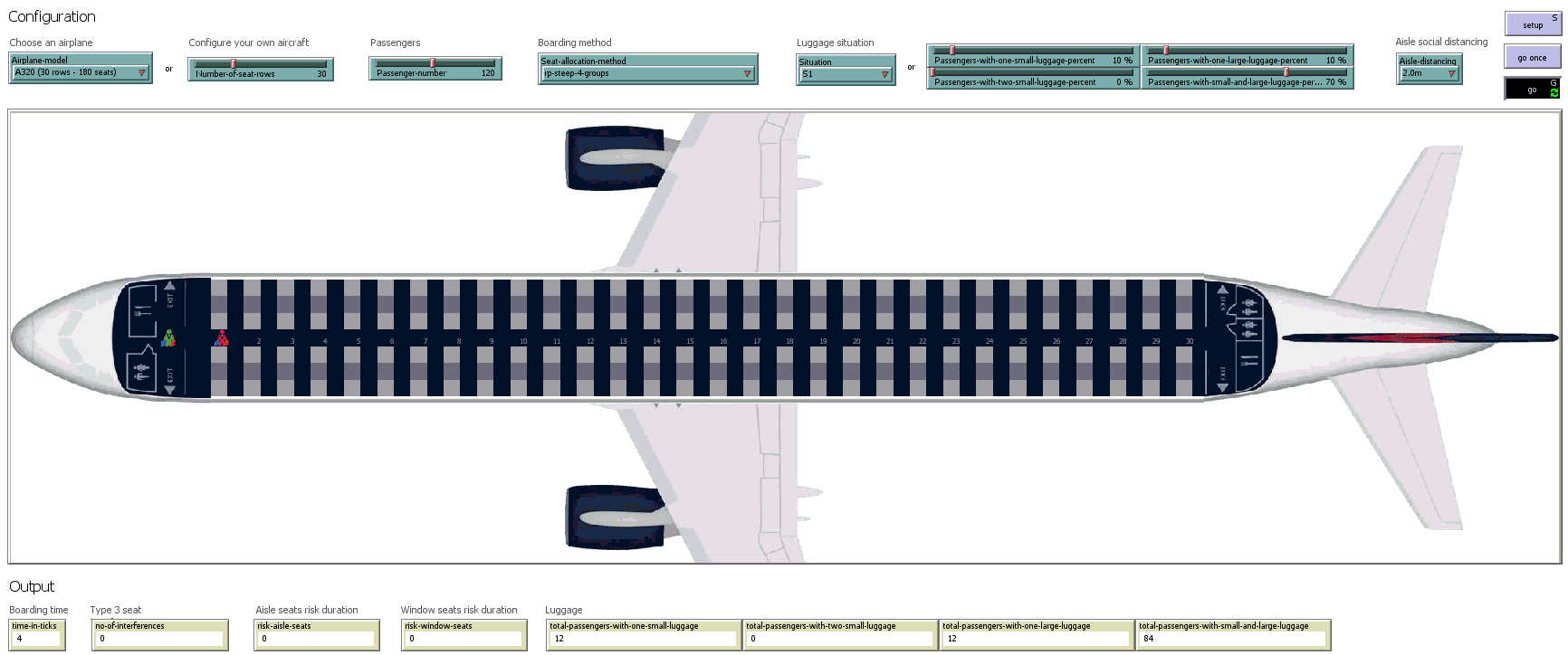

Supplement: S14 Fig — (GIF) [file pone.0242131.s014.gif]

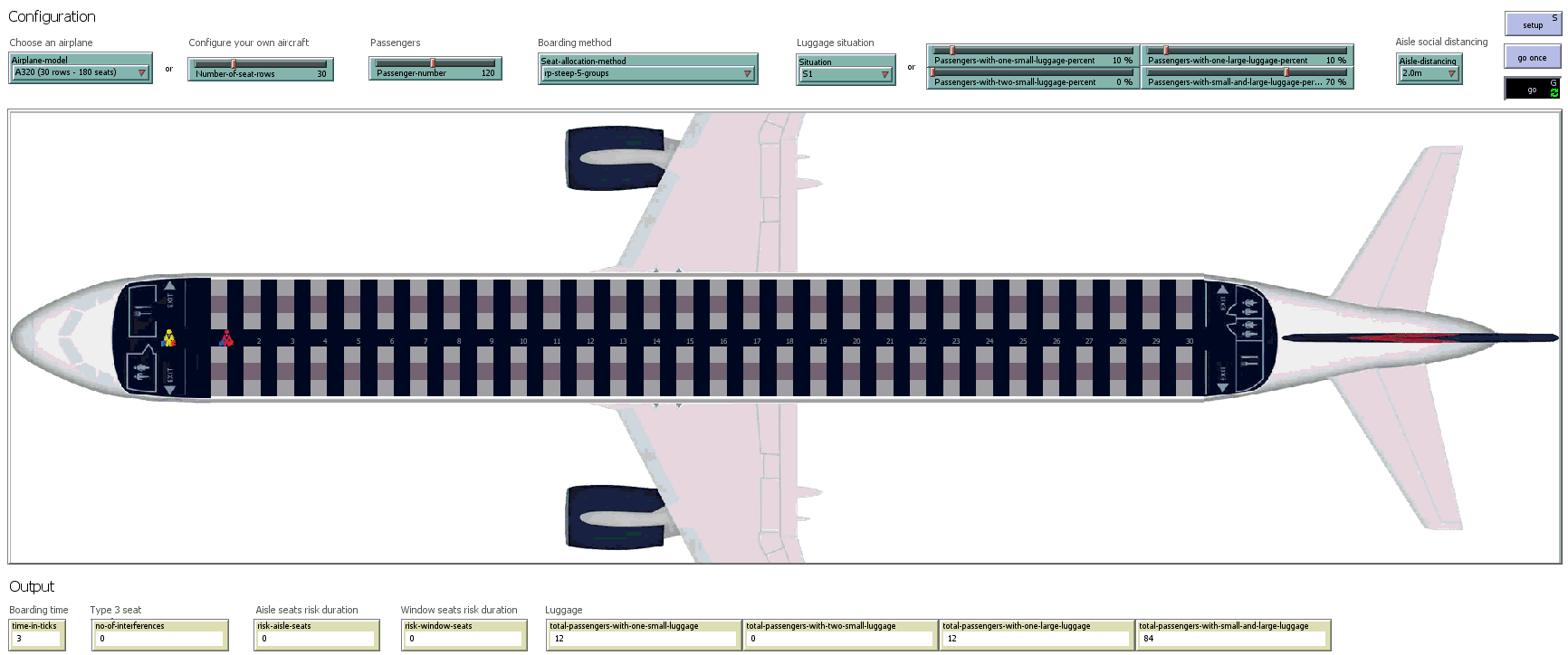

Supplement: S15 Fig — (GIF) [file pone.0242131.s015.gif]

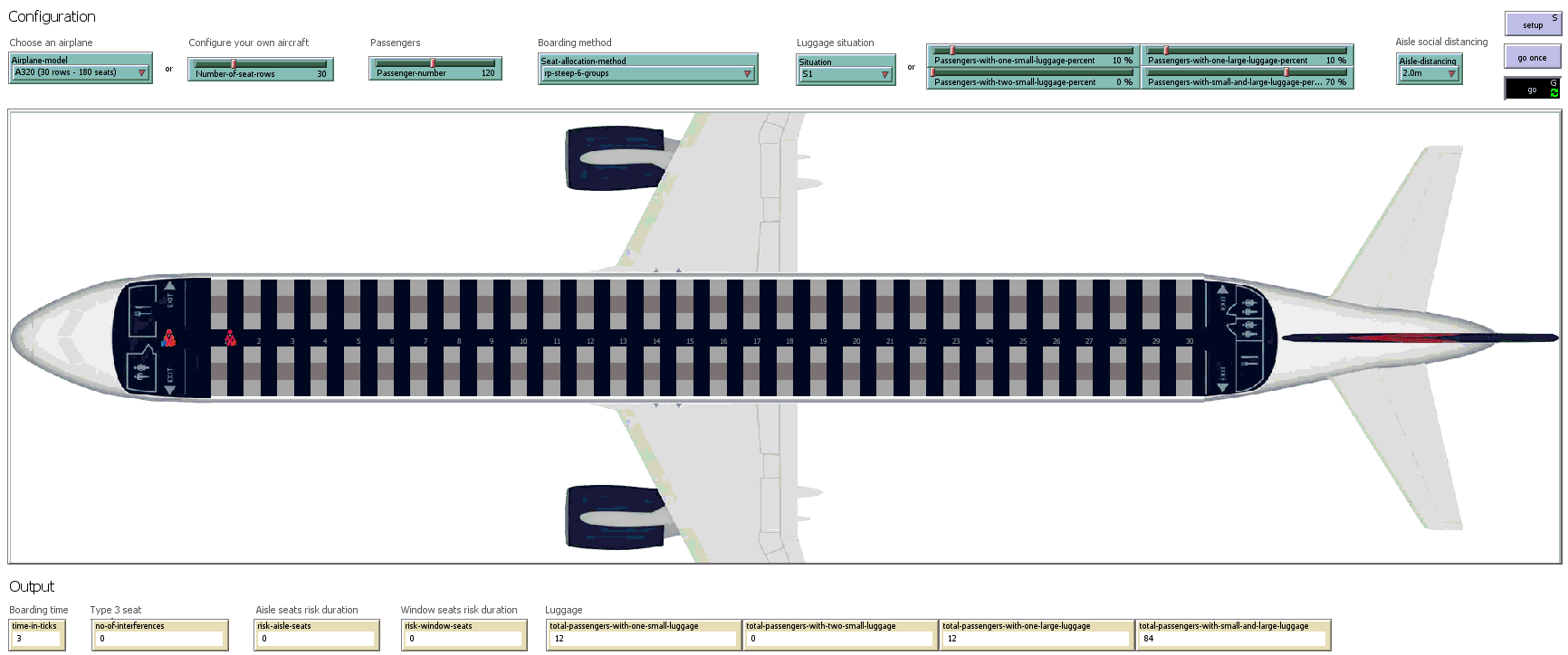

Supplement: S16 Fig — (GIF) [file pone.0242131.s016.gif]
